# Intercalated architecture of MA_2_Z_4_ family layered van der Waals materials with emerging topological, magnetic and superconducting properties

**DOI:** 10.1038/s41467-021-22324-8

**Published:** 2021-04-21

**Authors:** Lei Wang, Yongpeng Shi, Mingfeng Liu, Ao Zhang, Yi-Lun Hong, Ronghan Li, Qiang Gao, Mingxing Chen, Wencai Ren, Hui-Ming Cheng, Yiyi Li, Xing-Qiu Chen

**Affiliations:** 1 Shenyang National Laboratory for Materials Science, Institute of Metal Research, Chinese Academy of Sciences, 110016 Shenyang, People’s Republic of China; 2 School of Materials Science and Engineering, University of Science and Technology of China, 110016 Shenyang, People’s Republic of China; 3 School of Physics and Electronics, Key Laboratory for Matter Microstructure and Function of Hunan Province, Hunan Normal University, 410081 Changsha, People’s Republic of China; 4 Key Laboratory of Low-Dimensional Quantum Structures and Quantum Control of Ministry of Education, 410081 Changsha, People’s Republic of China; 5 Shenzhen Geim Graphene Center, Tsinghua-Berkeley Shenzhen Institute (TBSI), Tsinghua University, 518055 Shenzhen, People’s Republic of China

**Keywords:** Superconducting properties and materials, Magnetic properties and materials, Topological insulators

## Abstract

The search for new two-dimensional monolayers with diverse electronic properties has attracted growing interest in recent years. Here, we present an approach to construct MA_2_Z_4_ monolayers with a septuple-atomic-layer structure, that is, intercalating a MoS_2_-type monolayer MZ_2_ into an InSe-type monolayer A_2_Z_2_. We illustrate this unique strategy by means of first-principles calculations, which not only reproduce the structures of MoSi_2_N_4_ and MnBi_2_Te_4_ that were already experimentally synthesized, but also predict 72 compounds that are thermodynamically and dynamically stable. Such an intercalated architecture significantly reconstructs the band structures of the constituents MZ_2_ and A_2_Z_2_, leading to diverse electronic properties for MA_2_Z_4_, which can be classified according to the total number of valence electrons. The systems with 32 and 34 valence electrons are mostly semiconductors. Whereas, those with 33 valence electrons can be nonmagnetic metals or ferromagnetic semiconductors. In particular, we find that, among the predicted compounds, (Ca,Sr)Ga_2_Te_4_ are topologically nontrivial by both the standard density functional theory and hybrid functional calculations. While VSi_2_P_4_ is a ferromagnetic semiconductor and TaSi_2_N_4_ is a type-I Ising superconductor. Moreover, WSi_2_P_4_ is a direct gap semiconductor with peculiar spin-valley properties, which are robust against interlayer interactions. Our study thus provides an effective way of designing septuple-atomic-layer MA_2_Z_4_ with unusual electronic properties to draw immediate experimental interest.

## Abstract (web summary)

The discovery of a new two-dimensional van der Waals layered MoSi_2_N_4_ material inspires many attentions. Here, the authors report intercalation strategies to explore a much wider range of MA_2_Z_4_ family and predict amount of materials accessible to experimental verifications with emergent topological, magnetic or Ising superconductivity properties.

## Introduction

Monolayer two-dimensional (2D) materials have attracted tremendous interest for their unique electronic properties distinct from corresponding bulk phases, which show promising potential in a variety of fields such as energy storage and conversion^1,2^, nanoelectronics^3,4^, spintronics^5,6^, and superconductivity^7–16^. The electronic properties are strongly related to their geometric structures, which can be classified by the number of atomic layers (*n*).

**Fig. 1:**
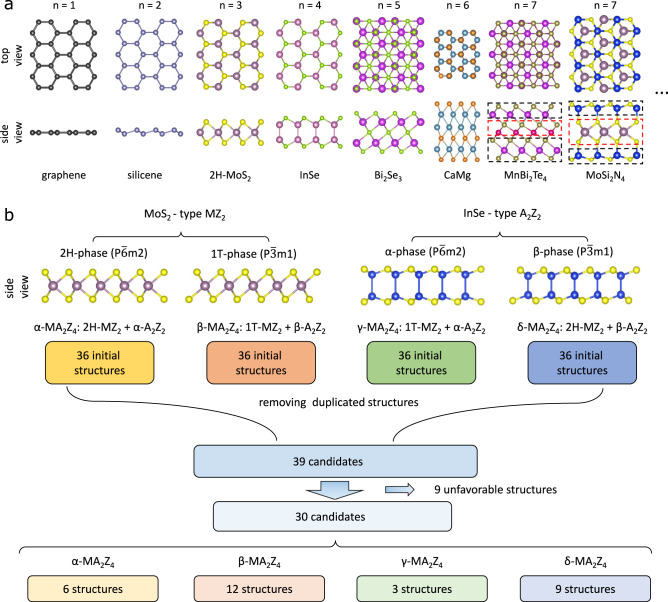
Geometric structures of known monolayer 2D materials and new MA_2_Z_4_ monolayer materials. **a** Top and side views of representative monolayers for *n* ≤ 7, all belonging to the SMR materials^17^. **b** Schematic illustration of the intercalation method that uses the structures of a MoS_2_-like monolayer (2*H*- and 1*T* phases) and those of an InSe-like monolayer (*α* and *β* phases) to construct the structures of a MA_2_Z_4_ monolayer.

Up to now, most of the discovered monolayer 2D materials have a thickness with *n* ≤ 7. Graphene and hexagonal boron nitride (*h*-BN) monolayer are famous six-membered ring (SMR) materials^17^ of *n* = 1, which have been a central topic for over a decade^18–26^. In the silicon counterpart of graphene, the hexagonal honeycomb tends to be buckled into a double-atomic layer structure, that is, *n* = 2 (see Fig. 1a)^27^. As for *n* = 3, monolayer transition-metal dichalcogenides (TMDs) are the most studied SMR materials^28,29^. In particular, 1${T}^{\prime}$-WTe_2_ monolayer is predicted to be a quantum spin Hall insulator^30,31^. Another popular system of *n* = 3 is the 2D ferromagnetic semiconductor CrI_3_, of which the magnetism can be manipulated by external electric fields and electrostatic dopings^32–34^. Among the family of *n* = 4, the monolayer group III chalcogenides have accepted growing interest. For instance, the semiconducting monolayer InSe exhibits extraordinary photocatalytic properties^35^. A strong electron–phonon coupling (EPC) can be obtained by an appropriate hole doping for this system, leading to interesting optical and transport properties^36,37^. The topological insulator Bi_2_Se_3_ and 2D ferromagnetic semiconductor CrGeTe_3_ are two representative systems of *n* = 5^38–40^. Recently, CaMg was found to have a sextuple layer (*n* = 6) structure^41^.

The exploration of monolayer 2D materials has been extended to septuple layer systems in recent years. For instance, a ferromagnetic semiconducting monolayer of MnBi_2_Te_4_ (Fig. 1a) can be obtained from its antiferromagnetic bulk phase, which has nontrivial topological properties^42–45^. More recently, we experimentally synthesized a new compound of septuple layer SMR material, that is, MoSi_2_N_4_ (see Fig. 1a), which has a bandgap of ~1.94 eV with excellent ambient stability^46^. The geometric structures of these materials can be related to other layered systems. For instance, the structure of the MnBi_2_Te_4_ monolayer can be viewed as an intercalated structure that is formed by inserting a single layer of the (111) plane MnTe (*n* = 2) into a Bi_2_Te_3_ monolayer (*n* = 5)^47^. Likewise, the MoSi_2_N_4_ monolayer can be built by intercalating a 2*H*-MoS_2_-type MoN_2_ layer (*n* = 3) into an *α*-InSe-type Si_2_N_2_ (*n* = 4).

As illustrated in Fig. 1a, we have summarized these known monolayer 2D SMR materials consisting of *n* = 1, 2, 3, 4, 5, 6, and 7 atomic layer thicknesses. With varying *n* number, compositions, and constituents, they will become richer in both structures and properties. However, the difficulties lie in how we effectively seek for more monolayer materials with unusual electronic properties. Here, we have proposed a general intercalated architecture approach to systemically construct the structures of MA_2_Z_4_ monolayers for the *n* = 7 family. We illustrate this scheme using density functional theory (DFT) calculations. In addition to reproducing the experimentally synthesized *α*_1_-MoSi_2_N_4_, *α*_1_-WSi_2_N_4_, and *β*_5_-MnBi_2_Te_4_ monolayer materials, we also predict 70, thermodynamically and dynamically, stable MA_2_Z_4_ monolayer materials, which exhibit diverse electronic properties including nontrivial topological properties, 2D ferromagnetism, Ising superconductivity, and robust electron valleys.

## Results

### Intercalated architecture approach

We construct the structures of hexagonal MA_2_Z_4_ monolayers by intercalating a MoS_2_-type MZ_2_ layer into an InSe-type A_2_Z_2_ monolayer, that is, the MZ_2_ layer is sandwiched between two AZ layers. We consider two types of phases for each constituent, that is, the 2*H* and 1*T* phases for the hexagonal MZ_2_ and the *α* and *β* phases for the A_2_Z_2_ monolayer. Each type of the combinations of MZ_2_ and A_2_Z_2_ involves one pair of the phases. We note that on each side of the MZ_2_ layer, there are three high-symmetry sites for both A and Z atoms in the AZ layers. Therefore, there are 36 configurations for each combination of MZ_2_ and A_2_Z_2_. As a result, there are 144 possible configurations for each compound. We reduce the number of structures to 39 by removing the duplicated structures based on a symmetry analysis. In addition, we found that, among them, nine configurations are energetically unfavorable. Therefore, we have considered 30 structures (Supplementary Table 1) for our first-principles calculations for each compound, which can be classified into four types, that is, *α*, *β*, *γ*, and *δ* respectively (see Fig. 1b).

As a benchmark, we first apply the scheme to MnBi_2_Te_4_, MoSi_2_N_4_, and WSi_2_N_4_. Our calculations found that the *β*_5_ phase has the lowest energy for MnBi_2_Te_4_, which is exactly the structure of a single MnBi_2_Te_4_ layer in the bulk phase^42–44,48^. For MoSi_2_N_4_ and WSi_2_N_4_, our results reveal that the *α*_1_ phase is the most stable structure, in agreement with our previous experiments^46^. Moreover, we have applied a structure prediction method RG^2^ to search for possible structures for MoSi_2_N_4_ and WSi_2_N_4_^49^. As a result, the predicted low-energy structures are within the 30 candidates. These results demonstrate the validity of our strategy in predicting monolayers with a septuple-atomic-layer structure.

### Energetics and stability of MA_2_Z_4_ monolayers

We now investigate the energetics and stability of MA_2_Z_4_ monolayers. We have performed calculations for MA_2_Z_4_ (M = elements of transition-metal groups IVB, VB, and VIB; A = Si, and Ge; and Z = N, P, and As). There are 54 compounds for this type of material, which have a number of valence electrons ranging from 32 to 34. Moreover, we have also carried out calculations for MA_2_Z_4_ monolayers (M = alkali-earth group elements Mg, Ca, and Sr and group IIB elements Zn, Cd, and Hg; A = Al and Ga; and Z = S, Se, and Te). There are 36 compounds in this family, which have a number of 32 valence electrons.

**Fig. 2:**
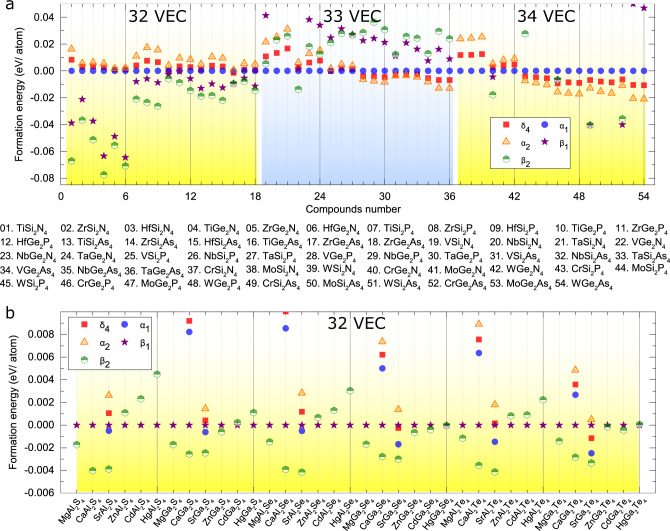
DFT-derived enthalpies of formation of MA_2_Z_4_ monolayers. Here, we only show the data for the five lowest-energy configurations. **a** and **b** are for *M* being the first transition-metal group elements and alkali-earth elements, respectively. In **a**, **b**, the enthalpies for each compound are relative to the total energies of the *α*_1_ and *β*_1_ structures, respectively. Note that VEC is the number of valence electrons for one chemical formula. For more details about enthalpies of formation refer to Supplementary Tables 2–7.

In Fig. 2a, b, we show the enthalpies of formation of the five lowest-energy structures for each compound, for which the total energies of the *α*_1_ and *β*_1_ phases are used as the reference, respectively. More information about the enthalpies of formation and structural properties can be found in Supplementary Tables 2–7. We found that the *β*_2_ phase has the lowest energy for all the 32-electron systems, with M being the transition-metal group elements. The trends for the systems with 33 and 34 valence electrons are different. For MSi_2_N_4_ (M = V, Nb, Ta, Cr, Mo, W), MGe_2_N_4_ (M = Nb, Ta, Mo, W), and MSi_2_P_4_ (M = Nb, Ta), the *α*_1_ phase is the lowest-energy structure, whereas for MSi_2_P_4_ (M = Cr, Mo, W), MGe_2_P_4_ (M = V, Nb, Ta, Cr, Mo, W), and M(Si/Ge)_2_As_4_ (M = V, Nb, Ta, Mo, W), the *α*_2_ phase is found to be energetically lower than the others. We further investigate their stability by performing phonon calculations, for which the results are summarized in Supplementary Fig. 1. Our calculations suggest that among the studied systems, with M being the transition-metal group elements, there are 48 compounds dynamically stable. The other six compounds, that is, *β*_2_-TiSi_2_N_4_, *β*_2_-TiGe_2_N_4_, *β*_2_-VGe_2_N_4_, *β*_2_-CrGe_2_N_4_, *β*_1_-CrSi_2_As_4_, and CrGe_2_As_4_, may be dynamically unstable since they exhibit imaginary phonon branches.

The monolayers based on the alkali-earth group and group IIB elements are in either the *β*_1_ or *β*_2_ phase. Twenty-four compounds are expected to be stable based on the phonon calculations (Supplementary Fig. 2). It should be mentioned that four of them, that is, *β*_1_-ZnAl_2_S_4_, *β*_1_-ZnGa_2_S_4_, *β*_1_-MgAl_2_S_4_, and *β*_1_-MgAl_2_Se_4_, can be found from the 2DMatPedia database, which was obtained by the high-throughput computational method^41^. This agreement again indicates the validity of our intercalation method in predicting structures of the MA_2_Z_4_ monolayers. Moreover, we have performed ab initio molecular dynamics simulations for selected systems, that is, *β*_2_-SrGa_2_Te_4_, *δ*_4_-VSi_2_P_4_, *α*_1_-TaSi_2_N_4_, and *α*_2_-WSi_2_P_4_ (Supplementary Fig. 3), whose electronic properties will be discussed below. Our results indicate that they are thermally stable. In all, we have predicted 72 new monolayer materials with a septuple-atomic-layer structure.

### Electronic properties

The intercalation method leads us to find a series of septuple layer MA_2_Z_4_ materials with diverse electronic properties, summarized in Supplementary Figs. 4–7, which are distinctly different from those of the constituents MZ_2_ and A_2_Z_2_. For instance, the 2*H*-NbN_2_ monolayer is a metallic ferromagnet, while *α*-Si_2_N_2_ is a semiconductor. However, the intercalated system *α*_1_-NbSi_2_N_4_ is surprisingly a ferromagnetic semiconductor (Supplementary Fig. 5). We found that the electronic properties of MA_2_Z_4_ can be classified according to the total number of valence electrons of these systems. The MA_2_Z_4_ nitrides with 32 valence electrons are semiconductors. However, their phosphides and arsenides with such a number of valence electrons are metallic, which is due to a much weaker band hybridization between the M and Z atoms in these systems than in the nitrides. Most of the MA_2_Z_4_ systems with 34 valence electrons are semiconductors, except for CrGe_2_N_4_, CrSi_2_As_4_, and CrGe_2_As_4_, which are ferromagnetic metals. In all, we found 21 semiconductors among the families of 32 and 34 valence electrons. We list the sizes of the bandgaps of all the semiconducting monolayers in Supplementary Table 2, which are from both the standard DFT and HSE hybrid functional (HSE06) calculations.

Besides, we have obtained many unexpected electronic properties for MA_2_Z_4_ monolayers, such as nontrivial topological properties, 2D ferromagnetism, Ising superconductivity, and robust electron valleys with spin-momentum locking. In the following, we will discuss them in more detail.

### Systems with 32 valence electrons

We first demonstrate that some of these systems exhibit nontrivial topological properties. We pay our attention to the family of MA_2_Z_4_ (M = Mg, Ca, Sr, Zn, Cd, and Hg; A = Al and Ga; and Z = S, Se, and Te). Most of them are semiconductors. The semiconducting nature can be understood based on the structural properties and the chemical bonding in this type of system. In the InSe-like structure of the A_2_Z_2_ monolayer, there is a covalent bond between the two A atoms. However, this bond is broken upon intercalating an MZ_2_ monolayer into the A_2_Z_2_ unit to form the intercalated system MA_2_Z_4_. Consequently, each atom tends to be charge compensated, which makes this system semiconducting. We have predicted a series of topological insulators in this family on the Perdew–Burke–Ernzerhof (PBE) level (Supplementary Table 3). For instance, our calculations show that *β*_2_-SrGa_2_Se_4_ is a zero-gap semiconductor (Supplementary Fig. 8). Inclusion of spin–orbit coupling (SOC) leads to a gap opening of ~68 meV and band inversion at Γ. Our calculations see gapless states in this system and found that it has a value of *Z*_2_ = 1. These results suggest that *β*_2_-SrGa_2_Se_4_ is a topological insulator. We further investigate its topological properties by using the advanced HSE hybrid functional method. As a result, it becomes topologically trivial.

**Fig. 3:**
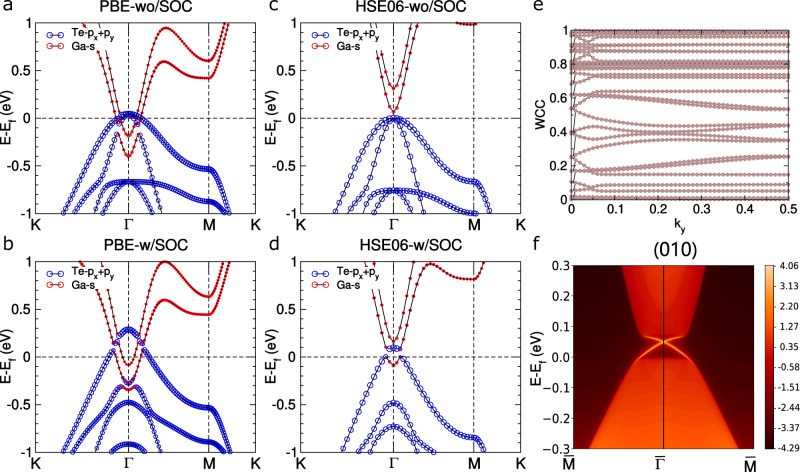
Topological properties of *β*_2_-SrGa_2_Te_4_. **a**, **b** The band structures from standard DFT calculations without and with SOC, respectively. **c**, **d** The results of HSE06 calculations without and with SOC, respectively. The bands are weighted by the orbital projections of Te and Ga. **e** The evolution of the Wannier charge centers (WCC) in the *k*_*z*_ = 0 plane. **f** Edge states of *β*_2_-SrGa_2_Te_4_ for the HSE06 calculations.

In contrast, we found that *β*_2_-SrGa_2_Te_4_ is topologically nontrivial by both the PBE and HSE06 methods. In Fig. 3a, we show the electronic bands from PBE calculations without SOC, which indicates that this system has a metallic band structure. The inclusion of SOC induces a gap opening near Γ, which results in a separation of the valence and conduction bands (see Fig. 3b). Similar to the case of *β*_2_-SrGa_2_Se_4_, we have *Z*_2_ = 1 and gapless edge states. We then investigate the topological property by performing HSE06 calculations without and with SOC, of which the results are shown in Fig. 3c, d, respectively. The calculation without SOC finds that there is a small direct bandgap at Γ. The inclusion of SOC induces a downward bending of the valence band at Γ, which signals the band inversion seen in the topological insulator Bi_2_Se_3_^38^. We further confirm this band inversion by the orbital-projected band structure shown in Fig. 3d. We have also calculated the evolution of the Wannier charge centers, which also gives *Z*_2_ = 1 (see Fig. 3e). Moreover, we have observed gapless edge states for *β*_2_-SrGa_2_Te_4_ (see Fig. 3f) based on the Wannier functions obtained from the HSE06 calculations, which again confirms that this system is topologically nontrivial.

### Systems with 33 valence electrons

These systems are in either the ferromagnetic state or the nonmagnetic state in this family. Among them, there are nine ferromagnetic systems, for which the magnetic configurations and the energetics are shown in Supplementary Fig. 9 and Supplementary Table 9, respectively. Our standard PBE calculations found that among them, NbSi_2_N_4_ and MGe_2_N_4_ (M = V, Nb, and Ta) monolayers show a half-metallic behavior (Supplementary Fig. 5), whereas VSi_2_Z_4_ (Z = N, P, and As) and VGe_2_Z_4_ (Z = P and As) exhibit a gapless semiconducting behavior^50,51^.

**Fig. 4:**
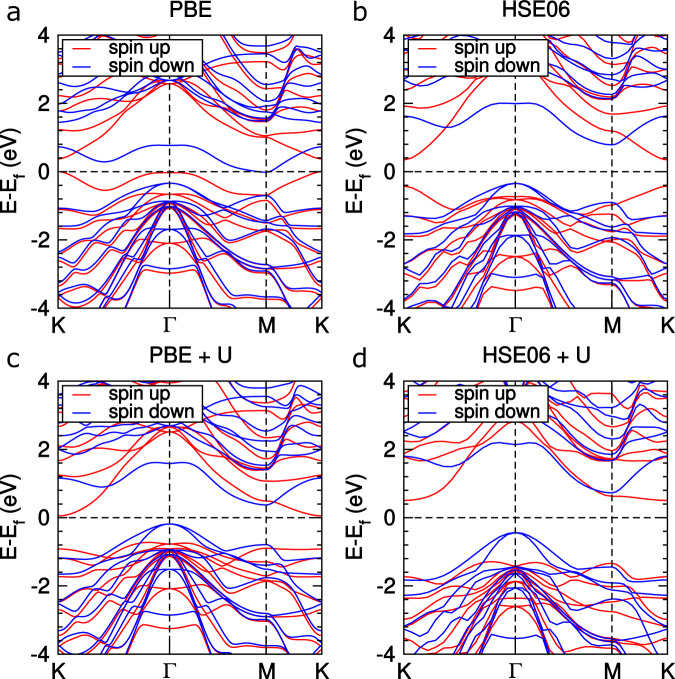
Band structures of ferromagnetic *δ*_4_-VSi_2_P_4_ under different methods. **a**, **b** Plots for PBE calculations with and without Hubbard *U*. **c**, **d** Corresponding plots for HSE06 calculations.

A close inspection of the PBE-derived band structure finds that the Fermi level of VSi_2_P_4_ touches both the valence band maximum (VBM) and conduction band minimum (CBM), which are the spin majority and minority states, respectively (Fig. 4a). In addition, we have calculated the magnetocrystalline anisotropy energy of VSi_2_P_4_, for which it is 56 μeV. This result indicates that its easy axis is out of plane. Furthermore, our HSE06 calculation reveals that it is nearly a direct bandgap semiconductor (Fig. 4b). We realized that the electron correlations related to the partially filled 3*d* orbitals of the transition-metal atoms may be important for the band structure of such a kind of system. We then estimate the effective Coulomb interaction (*U*) by using the linear-response method^52^, which gives *U* = 4 eV for the V-3*d* orbitals in VSi_2_P_4_. In Fig. 4c, we show the band structure of VSi_2_P_4_ from our PBE calculation with *U* (PBE + *U*). One can see that inclusion of the Hubbard *U* opens a gap of ~0.3 eV, which is then increased up to ~1.0 eV by the HSE06 calculation (Fig. 4d). Our calculations show that the HSE06 results are consistent with the PBE + *U* results that both predict a semiconducting behavior for the VSi_2_P_4_ monolayer. Moreover, we make a comparison of the band structures for the HSE06 calculations with and without Hubbard *U* and found that the sizes of the predicted bandgaps are comparable, although there are visible differences in details of the electronic bands near *K*. Based on these observations, we then performed HSE06 calculations for all the ferromagnetic systems. As a result, they all show a sizable gap ranging from 0.2 to ~1.0 eV (Supplementary Fig. 5).

We use the Heisenberg spin Hamiltonian to understand the magnetic interactions in the VSi_2_P_4_ monolayer. We extract the exchange interaction parameters between spins by fitting the total energies from our DFT calculations of various spin configurations to the Hamiltonian with the first nearest neighbors only, that is, *H* = − *J*∑_<*ij*>_*S*_*i*_⋅*S*_*j*_. We found that *J* is ~8 meV for *S* = 1. In fact, *J*_2_ is negligibly small (<0.1 meV) if we fit the Hamiltonian up to the second nearest neighbors. Based on the magnetic exchange parameters, we obtain a Curie temperature of ~90 K by performing Metropolis Monte Carlo simulations of the Heisenberg Hamiltonian (Supplementary Fig. 11).

**Fig. 5:**
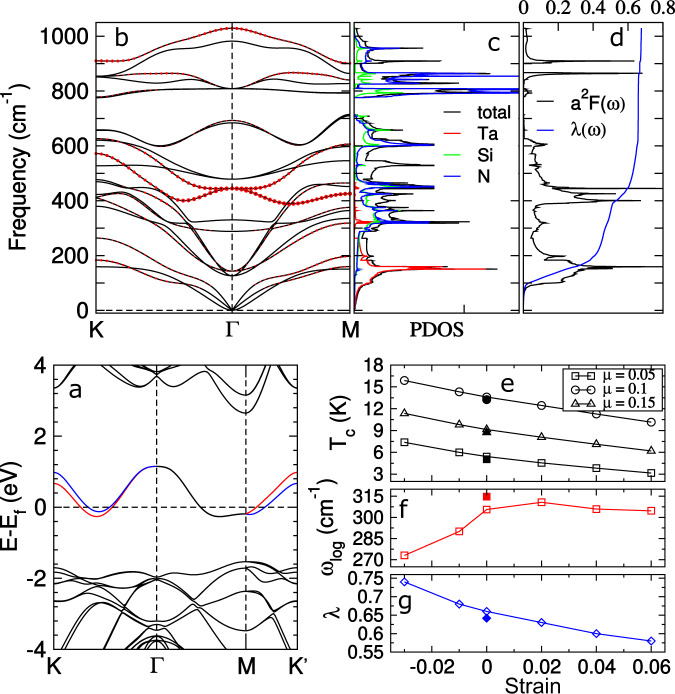
Ising superconductivity in *α*_1_-TaSi_2_N_4_. **a** Contrasting spin splitting at *K* and ${K}^{\prime}$ for *α*_1_-TaSi_2_N_4_ due to the SOC-induced Zeeman-like field. The red and blue lines represent different spin states. **b** Phonon dispersions and **c** phonon density of states of *α*_1_-TaSi_2_N_4_. **d** Eliashberg function *α*^2^*F*(*ω*) and the electron–phonon coupling strength *λ*(*ω*). Strain dependence of **e** the superconducting transition temperature (*T*_c_), **f** logarithmic average phonon frequency (*ω*_log_), and **g** electron–phonon coupling constant (*λ*). The solid symbols at the 0% strain denote the SOC-containing results.

The nonmagnetic systems of this family are metallic since they have one unpaired electron, which results in a half-filled electronic band (Supplementary Fig. 5). We note that among the systems, *α*_1_-TaSi_2_N_4_ has a special band structure, that is, it has a disentangled band contributed by the Ta-5*d* orbitals. Such a feature leads to the speculation of strong electron correlation in this system. Like in the case of VSi_2_P_4_, we have also carried out a linear-response calculation, which gives a value of ~2.5 eV for the *U* parameter of the Ta-5*d* orbitals. Then, we investigate the effects of Hubbard *U* on the band structure of TaSi_2_N_4_ and found that the half-filled band remains almost unchanged (Supplementary Fig. 12). Note that, in this system, the strong SOC along with the inversion symmetry breaking induces a large valley-contrasting spin splitting at *K* and *K*′. Such a band structure favors the type-I Ising superconductivity, as already observed in NbSe_2_^12^. Following this inspiration, we have calculated the phonon spectrum and Eliashberg function (*α*^2^*F*(*ω*)) as well as derived the EPC strength (*λ*) for *α*_1_-TaSi_2_N_4_ (Fig. 5). Using *λ* = 0.66 and the calculated logarithmic average phonon frequency of 305.58 cm^−1^, we have derived a superconducting transition temperature of *T*_c_ = 5.42–13.61 K via the Dynes modified McMillan formula with the effectively screened Coulomb repulsion constant of *μ* from 0.15 to 0.05. For *α*_2_-TaGe_2_P_4_, *T*_c_ is ~3.75 K for *μ* = 0.1 (Supplementary Fig. 13). Moreover, we have examined the effects of SOC and strain on *λ* and *T*_c_ of *α*_1_-TaSi_2_N_4_. We found that SOC has a minor effect on both *λ* and *T*_c_ (see Fig. 5g, e and Supplementary Fig. 13). We summarize our results of the strain effect (−3 to 6%) in Fig. 5e, which show that *T*_c_ varies nonlinearly with strain. *T*_c_ is only reduced by ~32% under a large strain up to 6%. While *T*_c_ is increased by ~24% under a compressive strain −3%. This trend indicates that the superconductivity in *α*_1_-TaSi_2_N_4_ is robust against strain, which favors the observation of Ising superconductivity in the epitaxially grown monolayer.

### Systems with 34 valence electrons

**Fig. 6:**
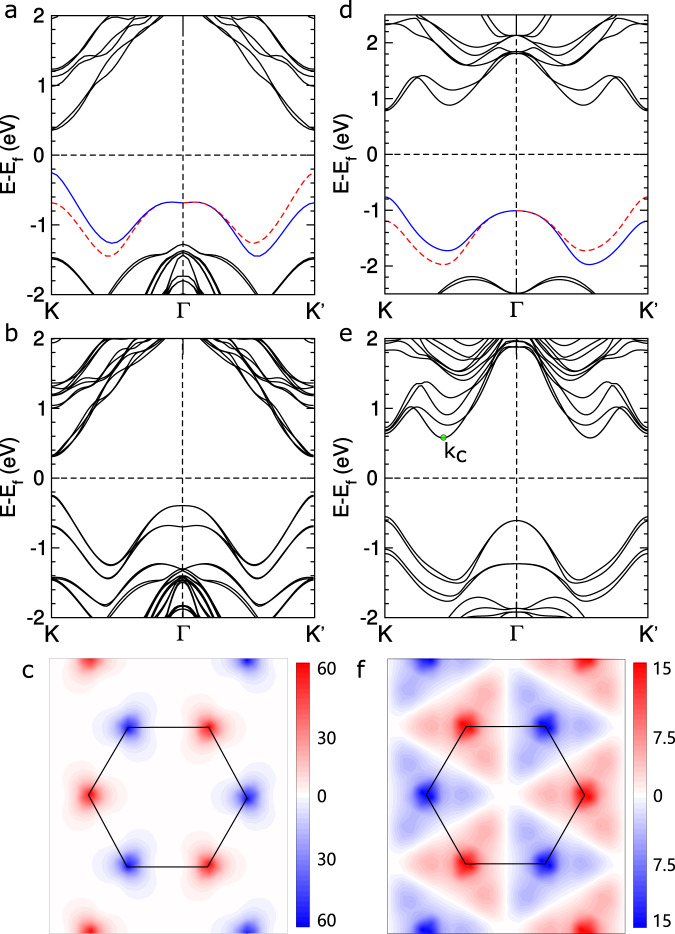
Comparison of electron valleys in between *α*_2_-WSi_2_P_4_ and 2*H*-WSe_2_. **a**, **d** Band structure for *α*_2_-WSi_2_P_4_ and 2*H*-WSe_2_ monolayers. The blue and red lines represent different spin states. **b**, **e** Band structure for the bilayer of *α*_2_-WSi_2_P_4_ and 2*H*-WSe_2_. *k*_c_ in **e** indicates the CBM. **d**, **f** Berry curvature of *α*_2_-WSi_2_P_4_ and 2*H*-WSe_2_ monolayers near the *K* and ${K}^{\prime}$ valleys. The black hexagons show the first Brillouin zones of the monolayers.

Among this family, we focus on Mo- and W-based MA_2_Z_4_ (A = Si and Ge and Z = N, P, and As) monolayers. The reason is that some of them even show better electronic properties than the dichalcogenide counterparts of Mo and W, that is, the TMDs monolayers. For the nitrides, that is, MSi_2_N_4_ and MGe_2_N_4_ (M = Mo and W), the lowest-energy structures are in the *α*_1_ phase, whereas for the phosphides and arsenides, the *α*_2_ structure becomes favorable. Despite this difference, the inversion symmetry is absent in both structures. Among these systems, MoSi_2_P_4_, MoSi_2_As_4_, WSi_2_P_4_, and WSi_2_As_4_ are predicted to be direct bandgap semiconductors by both the PBE and HSE06 calculations. One prominent feature of the band structure is that like the TMDs monolayers, there are electron valleys at *K* and ${K}^{\prime}$^53^. The strong atomic SOC in Mo and W induces large spin splittings at *K* and ${K}^{\prime}$. For *α*_2_-WSi_2_P_4_ (Fig. 6a), the spin splitting at *K* is ~0.4 eV, which is comparable to that of 2*H*-WSe_2_ monolayer^54,55^. In addition, the strong SOC together with the inversion symmetry breaking gives rise to spin-momentum locking at the valleys and a Berry curvature contrasting at the two valleys (see Fig. 6c). These features provide opportunities for exploring the spin-valley physics and manipulating their electronic properties via doping, interfacing, and layer stacking including Morie patterns^56,57^.

The unique structural and electronic structures of our systems show advantages over the TMDs layers in several aspects. First, the electron valleys in them are robust against interlayer interactions. In the case of the TMDs bilayers, by the layer hybridization, the conduction band at Γ is pushed up to a higher energy and the valence band at *k*_c_ is pushed down to a lower energy than that at *K* and ${K}^{\prime}$ (see Fig. 6e and Supplementary Fig. 15), resulting in a direct–indirect bandgap transition. Note that there is a large energy difference between the VBM at *K* (${K}^{\prime}$) and Γ for *α*_2_-WSi_2_P_4_. Moreover, the valence band and conduction band are dominated by the *d*-orbitals of W (Mo) (Supplementary Fig. 16), which is sandwiched by double-atomic AZ layers. Therefore, the VBM and CBM can be less affected by interfacing and layer stacking than those of the TMDs layers. In Fig. 6b, we show the band structure of the *α*_2_-WSi_2_P_4_ bilayer. One can see that the nature of the direct bandgap is maintained well upon the layer stacking. In addition, we found that *α*_2_-WSi_2_P_4_ exhibits a large hole mobility of up to 460 cm^2^ V^−1^ s^−1^ and an electron mobility of about 150 cm^2^ V^−1^ s^−1^ (see Supplementary Fig. 17 and Supplementary Table 8). These values are ~150% of those for the 2*H*-WSe_2_ monolayer^58,59^, indicating that *α*_2_-WSi_2_P_4_ may have better electronic transport properties than the TMDs monolayers.

In summary, we have presented an intercalation approach to construct septuple-atomic-layer MA_2_Z_4_ monolayers. We have illustrated this strategy by performing first-principles calculations for systems with 32–34 valence electrons. Our calculations predict that among 90 candidates, 72 compounds are both thermodynamically and dynamically stable. The systems with 32 and 34 valence electrons are mostly semiconductors, whereas those with 33 valence electrons are either nonmagnetic metals or ferromagnetic semiconductors. In addition, we found that these systems exhibit a number of novel electronic properties. In particular, *β*_2_-SrGa_2_Te_4_ is found to be a topological insulator by both the PBE and HSE06 calculations. In addition, our study finds that among the family of the systems with 33 valence electrons, the V-based MA_2_Z_4_ monolayers are basically ferromagnetic semiconductors as revealed by our HSE06 calculations. As for the nonmagnetic metals like *α*_1_-TaSi_2_N_4_ in this family, the inversion symmetry breaking and the strong SOC lead to a valley-contrast spin splitting in the half-filled band with out-of-plane spin polarizations. We further predict that such features favor the type-I Ising superconductivity in *α*_1_-TaSi_2_N_4_. Moreover, our study reveals that *α*_1_-WSi_2_P_4_ not only has a direct bandgap with robust electron valleys against layer interactions but also shows superior electronic transport properties compared to the TMDs monolayer WSe_2_. Finally, we would like to emphasize that our currently proposed intercalated architecture approach can be indeed extended to MA_2_Z_4_ monolayer materials with M for late transition metal elements, such as MnBi_2_Te_4_ for which our current calculations also correctly capture the agreements to experiments. Furthermore, it can be generalized to a wider way. For instance, *n*=7 MA_2_Z_4_ monolayer materials can be constructed by intercalating *n* = 2 silicene-like monolayer into *n* = 5 Bi_2_Se_3_-like monolayer and, we can also even combine *n* = 3 MoS_2_-like monolayer and *n* = 5 Bi_2_Se_3_-like monolaye to form new type *n* = 8 monolayer materials, and so on. Our study thus provides an effective way of designing septuple layer systems with unusual electronic properties.

## Methods

### Electronic and phononic band structures

First-principles calculations were performed by applying the Vienna ab initio simulation package (VASP)^60,61^, and the PBE exchange-correlation functional was used to calculate the enthalpy of formation and band structure. To further get a more accurate bandgap, a hybrid function (HSE06) was applied. The plane-wave energy cutoff was adopted to be ranging from 250 eV (MgGa_2_Te_4_) to 500 eV (MoSi_2_N_4_). The 2D Brillouin zone was sampled by a 15 × 15 *k*-mesh for the self-consistent calculations. Structural relaxations were done with a threshold of 10^−3^ eV Å^−1^ for the residual force on each atom. The energy convergence criteria were set as 10^−6^ eV. To minimize the interactions between the monolayer and its periodic images, a vacuum of 20 Å was used for all the calculations.

Phonon dispersions were obtained using density functional perturbation theory and Phonopy package^62^. We used a large supercell (either a 4 × 4 supercell or a 5 × 5 supercell) for the force calculations.

### EPC and superconductivity

For metallic materials, the EPC constant *λ* is given by^63^

1 $$\lambda =2\int{\mathrm{d}}\omega {\alpha }^{2}F(\omega )/\omega$$

where *α*^2^*F*(*ω*) is the Eliashberg function, which is defined as

2 $${\alpha }^{2}F(\omega )=\frac{1}{2\pi N\left({\epsilon }_{{\rm{F}}}\right)}\mathop{\sum}\limits _{{\boldsymbol{q}}\nu }\delta (\omega -{\omega }_{{\boldsymbol{q}}\nu })\frac{{\gamma }_{{\boldsymbol{q}}\nu }}{\hslash {\omega }_{{\boldsymbol{q}}\nu }},$$

where *N*(*ϵ*_*F*_) is the density of states at the Fermi level, *ω*_***q****ν*_ is the phonon frequency of the mode *ν* at wavevector ***q*** and *γ*_***q****ν*_ is the phonon linewidth or lifetime.

The Eliashberg function *α*^2^*F*(*ω*) is used to calculate logarithmic average phonon frequencies by $\omega_{\mathrm{log}} = {\mathrm{exp}} \left[\frac{2}{\lambda }\int_{0}^{\infty }\frac{{\mathrm{d}}\omega }{\omega }{\alpha }^{2}F (\omega ){\mathrm{log}}\,\omega \right]$. We use the Allen–Dynes-modified McMillan formula to estimate the superconducting transition temperature^64^, that is, ${T}_{\mathrm{c}}=\frac{\omega_{\mathrm{log}}}{1.2}{\mathrm{exp}} \left[\frac{-1.04 ( 1+ \lambda )}{\lambda -{\mu }^{* } (1 + 0.62\lambda )}\right]$.

The above calculations were performed using the Quantum-ESPRESSO package with ultrasoft pseudopotentials and local-density approximation exchange-correlation functional^52,65^. For *α*_1_-TaSi_2_N_4_, the kinetic energy cutoff and charge density cutoff of the plane-wave basis are 60 and 480 Ry, respectively. A 32 × 32 *k*-mesh with Marzari–Vanderbilt cold smearing of 0.02 Ry is used for self-consistent calculations. A 4 × 4 *q*-mesh is used to obtain dynamic matrix and EPC constant, respectively. For *α*_2_-TaGe_2_P_4_, a kinetic energy cutoff of 80 Ry and a charge density cutoff of 640 Ry are used. A 36 × 36 *k*-mesh and 6 × 6 *q*-mesh are used to calculate EPC constant and the superconducting *T*_c_.

### Carrier mobility

The intrinsic carrier mobility *μ* of 2D materials was derived based on the deformation potential (DP) approximation^66^

3 $${\mu }_{\mathrm{2D}}=\frac{2e{\hslash }^{3}C}{3{k}_{{\rm{B}}}T{\left|{m}^{* }\right|}^{2}{E}_{1}^{2}},$$

where *C* is the elastic modulus defined as $\left[{\partial }^{2}E/\partial {\delta }^{2}\right]/{S}_{0}$, m* is the effective mass at the CBM or VBM, and *T* is the temperature. Here, room temperature *T* = 300 K was used. *E*_1_ is the DP constant defined as ${{\Delta }}E/\left({{\Delta }}l/{l}_{0}\right)$, where Δ*E* is the change of the eigenvalue at CBM or VBM and Δ*l* is the lattice dilation along deformation direction.

### Calculation of the enthalpy of formation

The enthalpy of formation of a MA_2_Z_4_ monolayer (per atom) can be expressed as:

4 $${E}_{\mathrm{f}}=\{{E}_{{\rm{tot}}}-\left({E}_{{\mathrm{M}}}+2{E}_{{\rm{A}}}+4{E}_{{\rm{Z}}}\right)\}/7,$$

where *E*_tot_ is the total energy of the system, and *E*_M_, *E*_A_, and *E*_Z_ are the ground state total energies of the elementary crystals of M, A, and Z, respectively.

### Calculations of Berry curvature and the *Z*_2_ topological invariant

The Berry curvature of a 2D material with *n* bands can be defined as^67–69^:

5 $${{{\Omega }}}_{z}({\bf{k}})={\nabla }_{{\bf{k}}}\times i\langle {u}_{n,{\bf{k}}}| {\nabla }_{{\bf{k}}}{u}_{n,{\bf{k}}}\rangle ,$$

where *u*_*n*,**k**_ is the lattice periodic part of the Bloch wave functions. Our calculations were performed using the Wannier90 package^70^, which constructs *u*_*n*,**k**_ via an ab initio tight-binding method on the basis of maximally localized Wannier functions.

The values of the topological invariant *Z*_2_ were obtained by calculating the Wannier charge centers^71^. The edge states were obtained using an iterative Green functions method^72^. The above calculations were based on the ab initio tight-binding parameters from the Wannier90 calculations.

## Supplementary information

Supplementary Information

## Data Availability

In our Supplementary Materials, we have already represented all necessary data. The further data that support the findings of this study are available from the corresponding authors upon request.
