# Supplementary material for: Intercalated architecture of MA2Z4 family layered van der Waals materials with emerging topological, magnetic and superconducting properties
Source: Nat Commun. 2021 Apr 21;12:2361. doi: 10.1038/s41467-021-22324-8 (PMC8060390; doi:10.1038/s41467-021-22324-8)
Supplement: Supplementary file 1 — Supplementary Information [file 41467_2021_22324_MOESM1_ESM.pdf]

Supplementary Materials

Intercalated architecture of MA_2Z_4 family layered van der Walls materials with emerging topological, magnetic and superconducting properties

Lei Wang,[1,2] Yongpeng Shi,[1,2] Mingfeng Liu,[1,2] Ao Zhang,[3] Yi-Lun Hong,[1,2] Ronghan Li,[1,2] Qiang Gao,[1,2] Mingxing Chen,[3,*] Wencai Ren,[1,2] Hui-Ming Cheng,[1,2,4] Yiyi Li,[1,2] and Xing-Qiu Chen[1,2,*]

[1]*Shenyang National Laboratory for Materials Science, Institute of Metal Research, Chinese Academy of Science, 110016 Shenyang, Liaoning, P. R. China*

[2]*School of Materials Science and Engineering, University of Science and Technology of China, Heifei 230026, P. R. China*

[3]*School of Physics and Electronics, Hunan Normal University, Key Laboratory for Matter Microstructure and Function of Hunan Province, Key Laboratory of Low-Dimensional Quantum Structures and Quantum Control of Ministry of Education, Changsha 410081, P. R. China*

[4]*Shenzhen Geim Graphene Center, Tsinghua-Berkeley Shenzhen Institute (TBSI), Tsinghua University, 1001 Xueyuan Road, Shenzhen 518055, P. R. China*

[*] Corresponding author. E-mail: xingqiu.chen@imr.ac.cn; mxchen@hunnu.edu.cn

This PDF file includes:

Supplementary methods (Page 2)

Supplementary Figs. 1 to 18 (Page 3-15)

Supplementary Tables 1 to 10 (Page 16-24)

References

Supplementary methods

We derive the magnetic exchange constant for the nearest neighboring V atoms using the total energies obtained from DFT calculations for different magnetic configurations (see Supplementary Fig. 10).

In these configurations, there are FM and/or AFM couplings between the magnetic V atoms. In the figure, the energies due to the FM couplings between the first and second nearest neighboring V atoms are respectively denoted by $E_{F,1}$ and $E_{F,2}$. Likewise, the AFM couplings between the nearest neighboring V atoms is denoted by $E_{A,1}$.

The total energies of the ferromagnetic (FM) state with respect to the nonmagnetic (NM) can be written as

$$E_{FM} - E_{NM} = 3E_{F1} + E_{F2} \tag{1}$$

For the antiferromagnetic (AFM) state, we have

$$E_{AF} - E_{NM} = E_{F1} + 2E_{A1} + E_{F2} \tag{2}$$

.

Then, we obtain J = (E_{FM}-E_{AF})/(4S^2) by making use of the Heisenberg spin Hamiltonian.

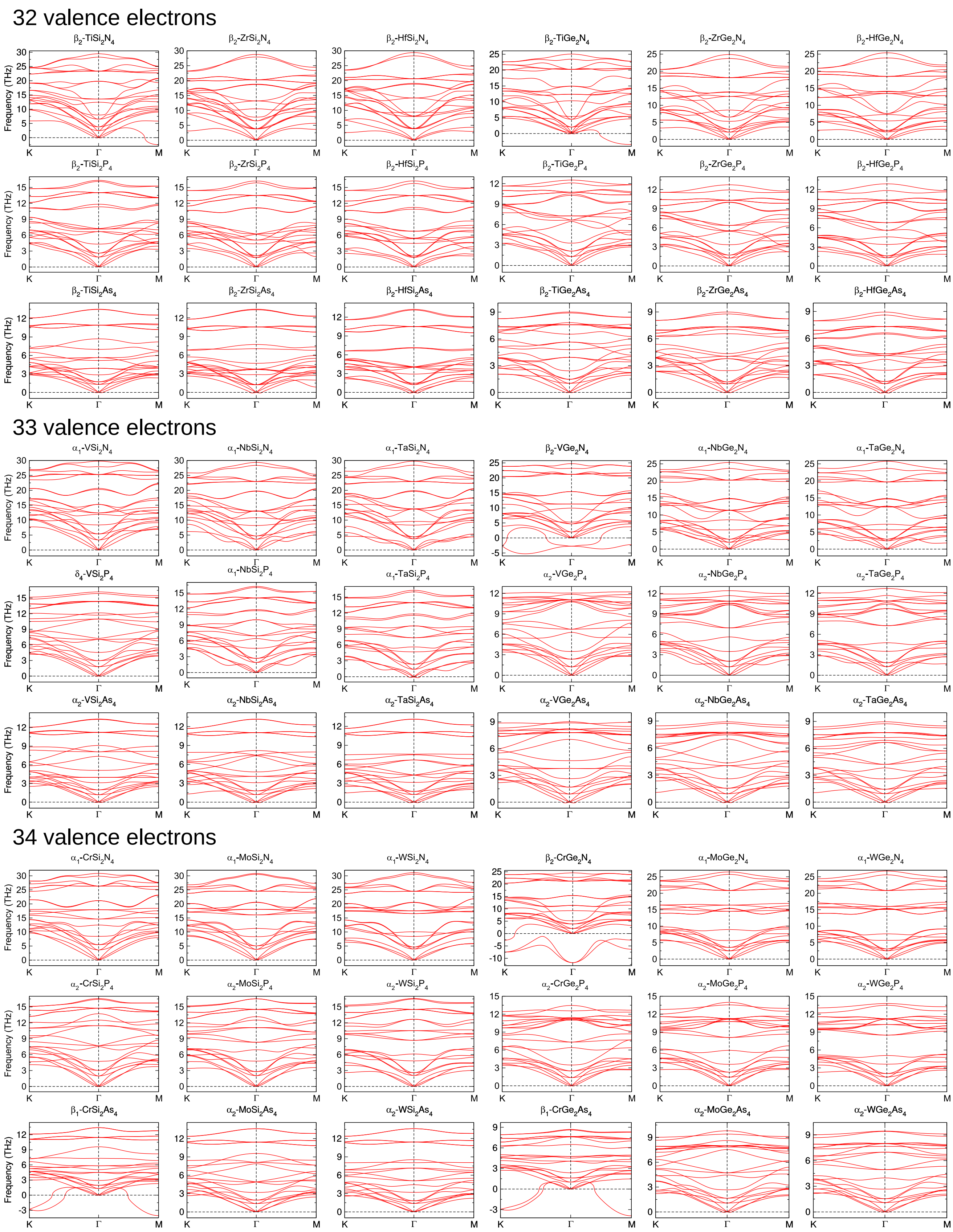

Supplementary Fig. 1. Phonon spectrum of MA_2Z_4 monolayers listed in **Supplementary Table 2**.

32 valence electrons

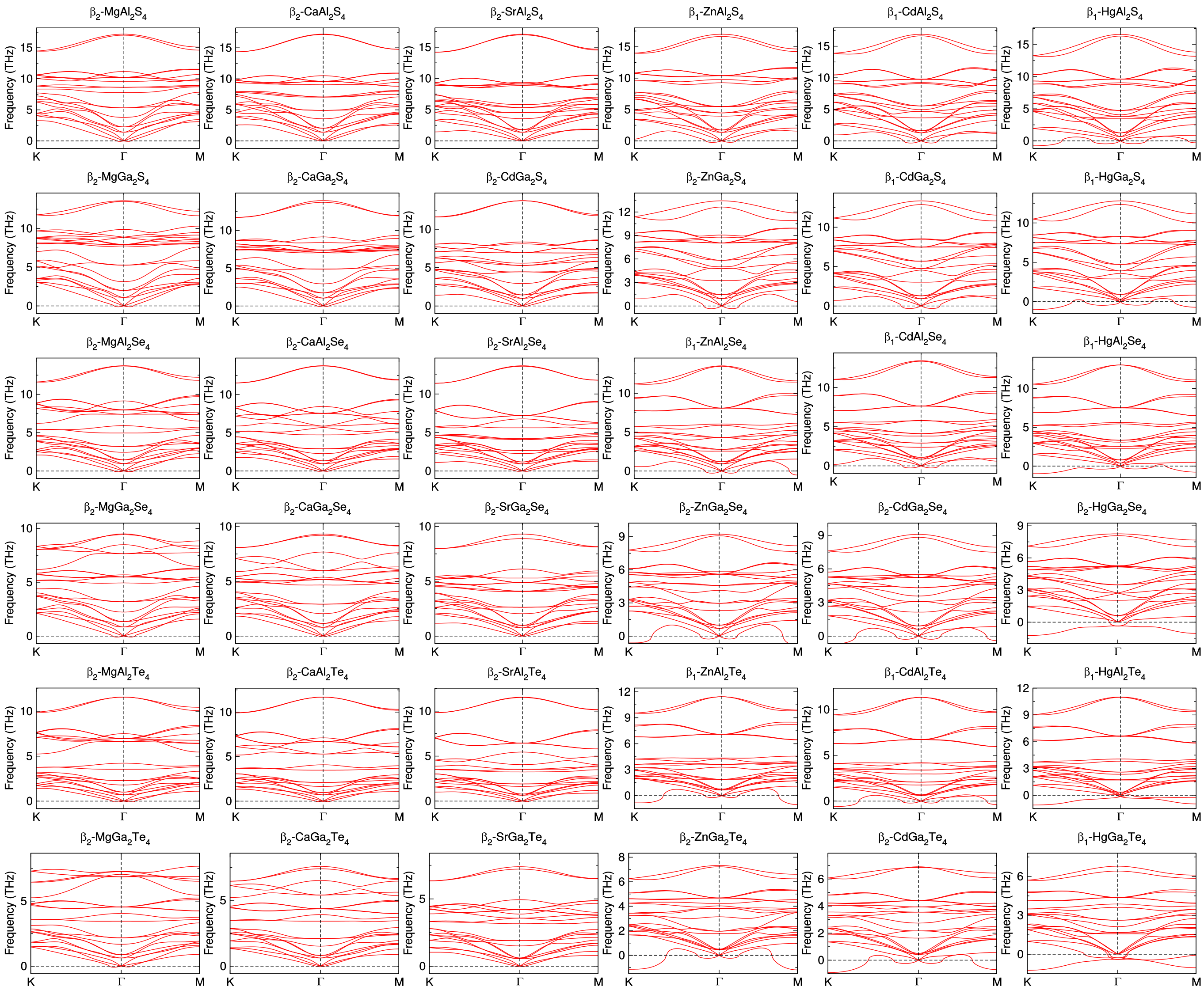

Supplementary Fig. 2. Phonon spectrum of MA_2Z_4 monolayers listed in **Supplementary Table 3**.

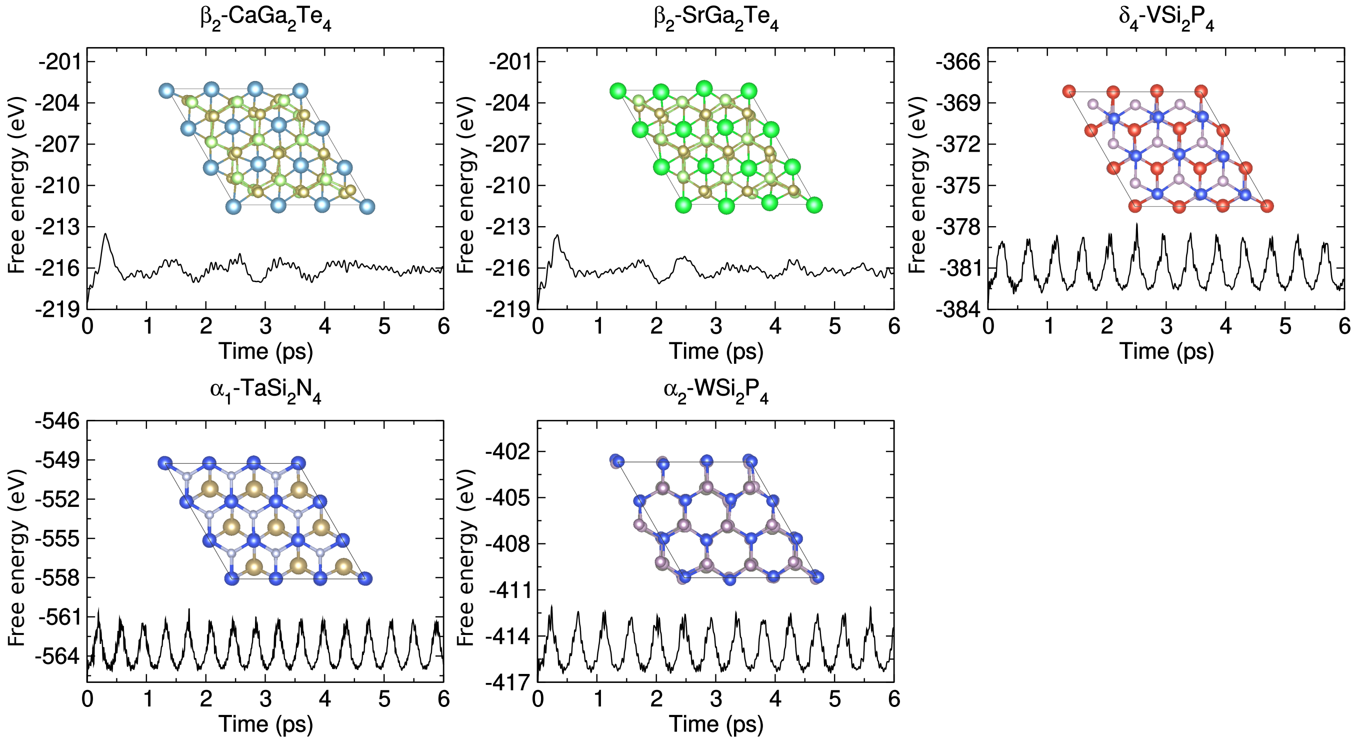

Supplementary Fig. 3. (color online) Variation of free energy for β_2-$CaGa_2Te_4$, β_2-$SrGa_2Te_4$, δ_4-VSi_2P_4, α_1-$TaSi_2N_4$ and α_2-WSi_2P_4 monolayer at 6 ps during AIMD simulation at 300 K.

32 valence electrons

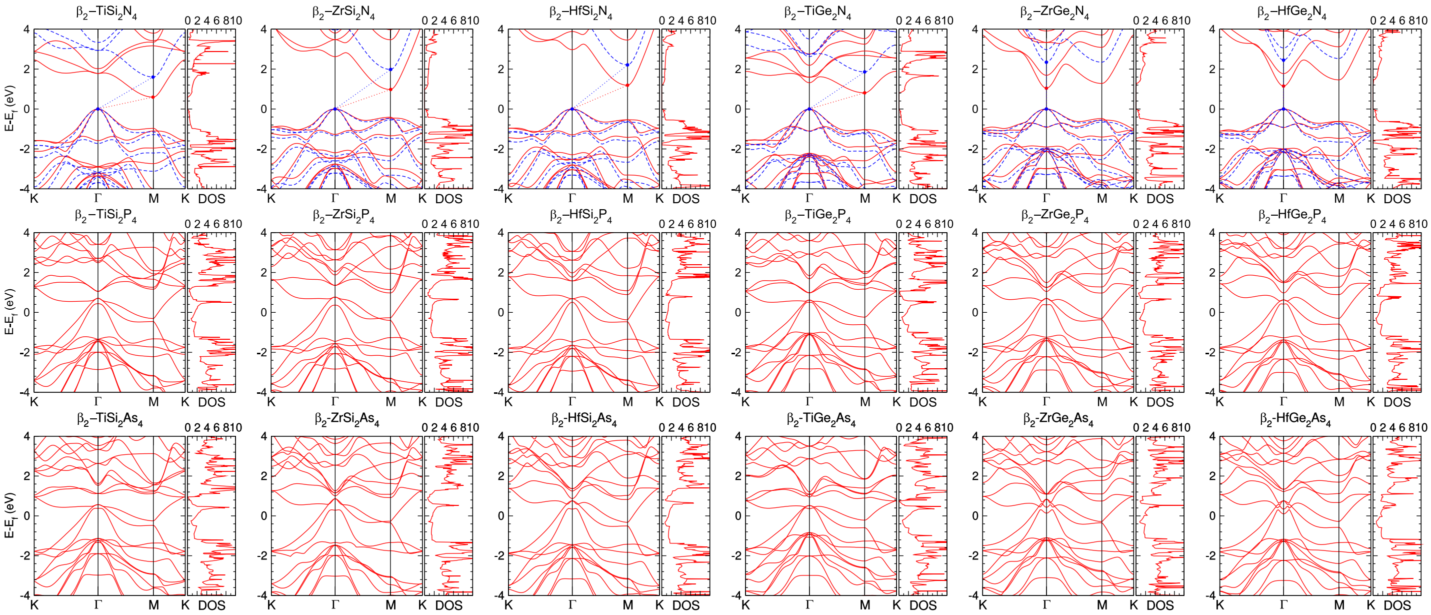

Supplementary Fig. 4. (color online) Electronic structures of MA_2Z_4 monolayers with 32 valence electrons listed in **Supplementary Table 2**. For semiconductors, the PBE and HSE bands are shown in red solid and blue dash lines, respectively.

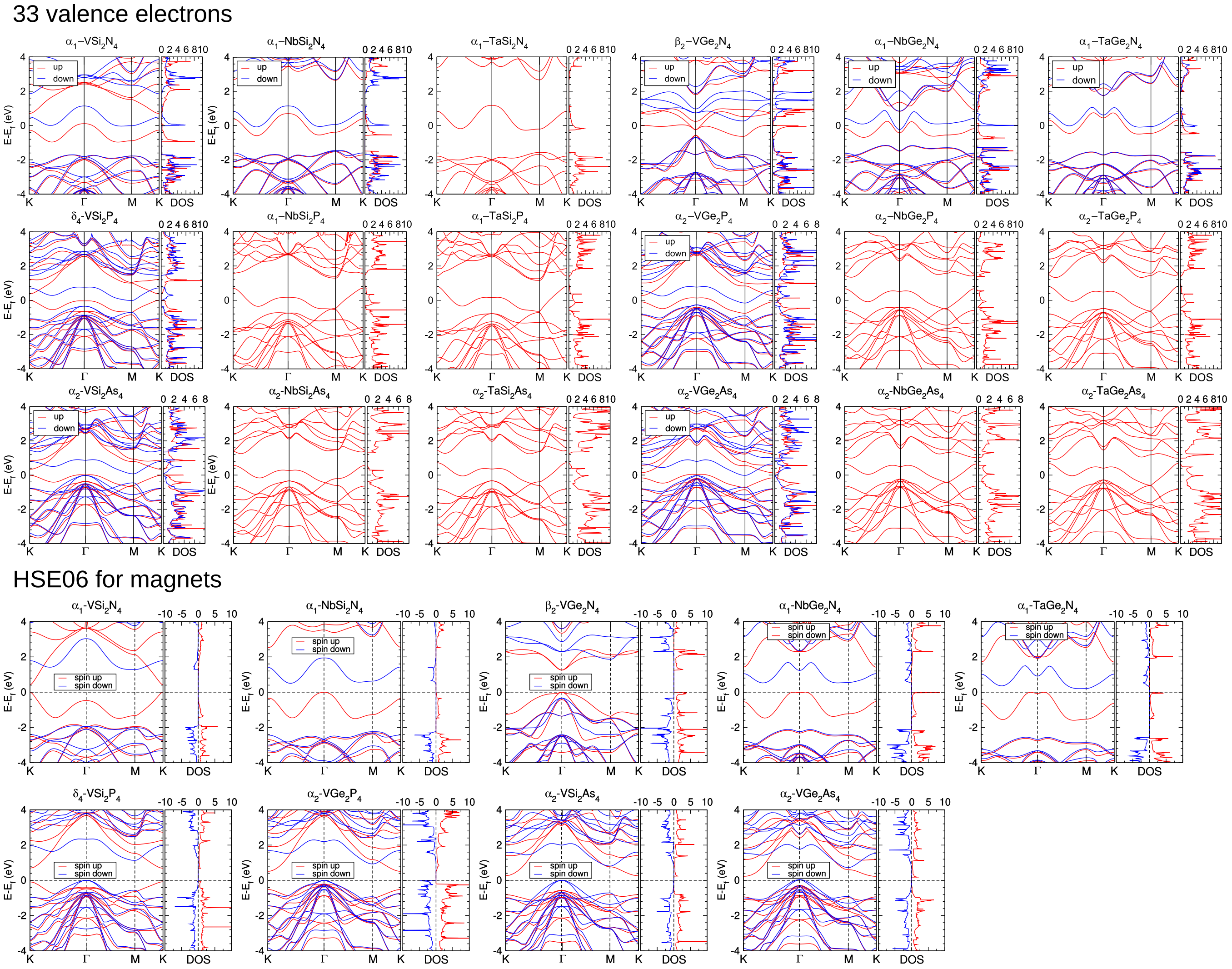

Supplementary Fig. 5. (color online) Electronic structures of MA_2Z_4 monolayers with 33 valence electrons listed in **Supplementary Table 2**. For ferromagnetic compounds, the red and blue solid lines represent the spin majority and minority bands, respectively.

34 valence electrons

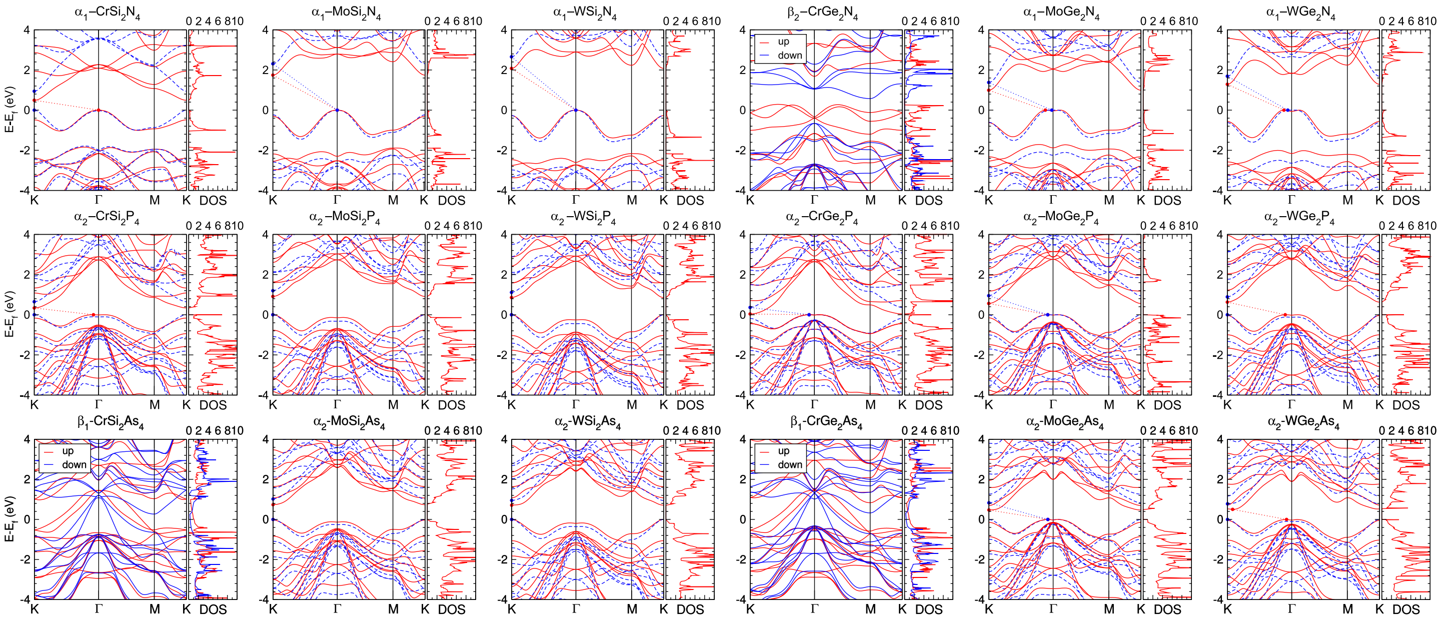

HSE06 for magnets

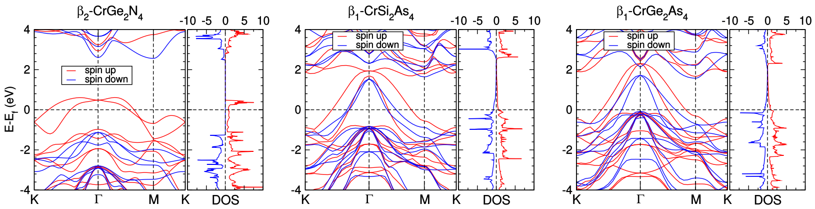

Supplementary Fig. 6. (color online) Electronic structures of MA_2Z_4 monolayers with 34 valence electrons listed in **Supplementary Table 2**. For semiconductors, the PBE and HSE bands are shown in red solid and blue dash lines, respectively. For ferromagnetic compounds, the red and blue solid lines represent the spin majority and minority states, respectively.

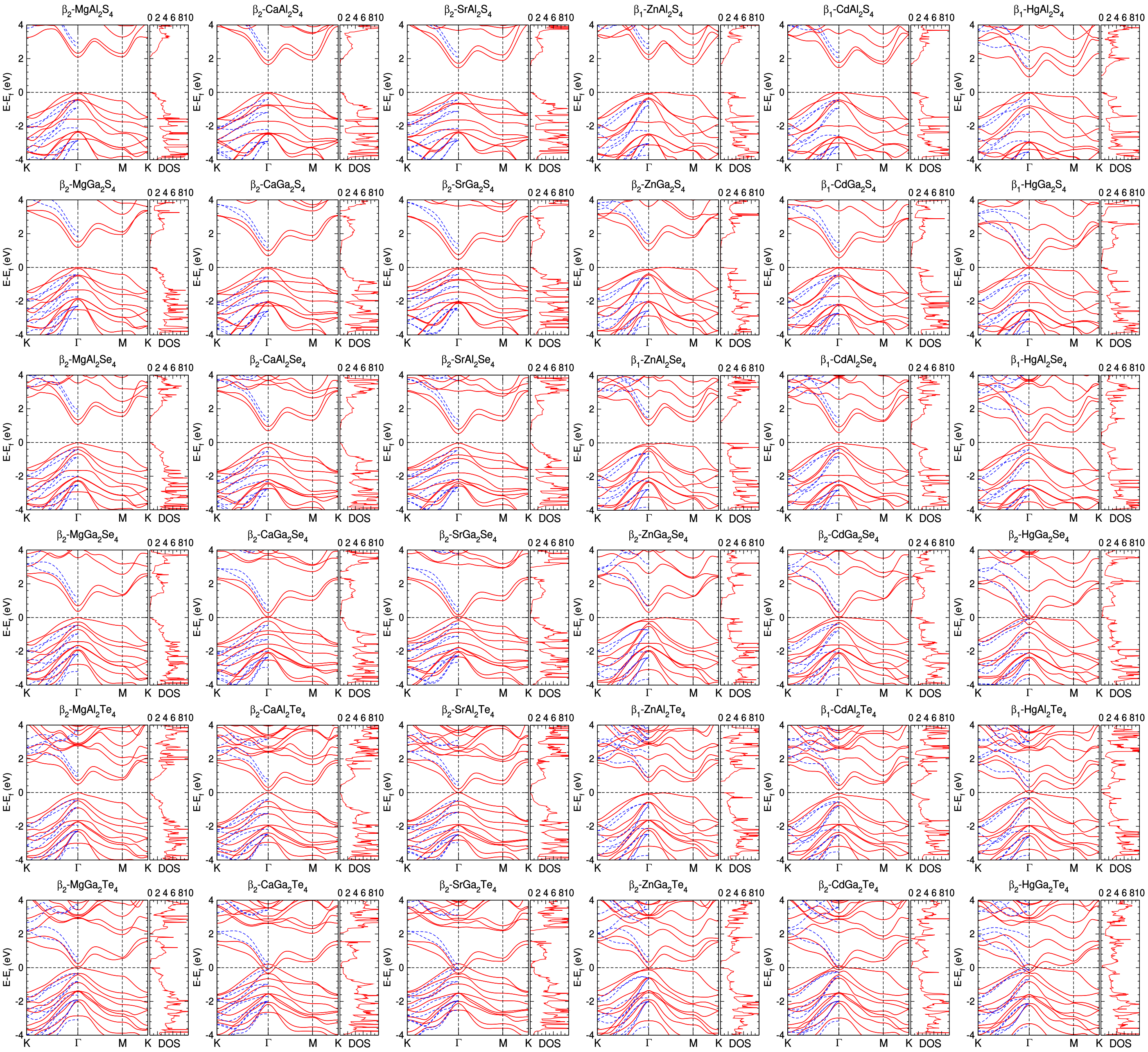

Supplementary Fig. 7. (color online) Electronic structures of MA_2Z_4 monolayers listed in **Supplementary Table 3**. The PBE and HSE bands are shown in red solid and blue dash lines, respectively.

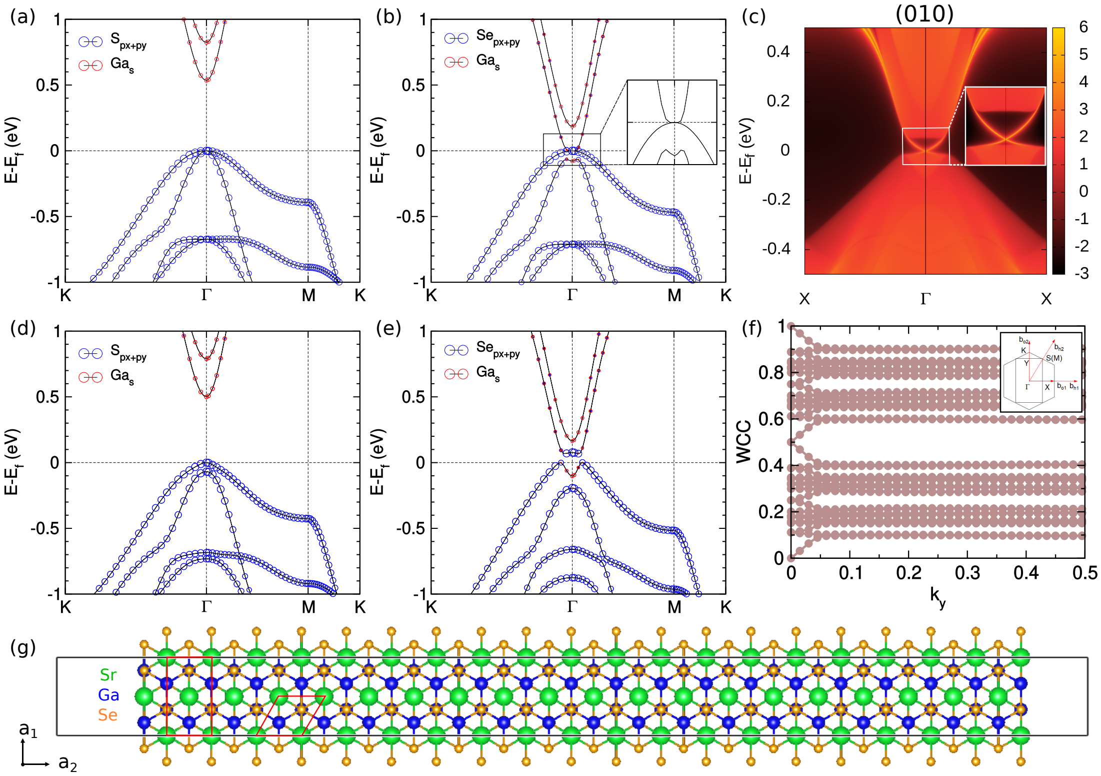

Supplementary Fig. 8. (color online) (a),(d) Band structures for β_2-$SrGa_2S_4$ without and with SOC, respectively. (b) and (e) Band structures for β_2-$SrGa_2Se_4$ without and with SOC respectively. The insert in (b) shows the zoom-in band degeneracy near the Fermi level. (c) Edge states along (010) and the insert shows the gapless edge states. (f) The evolution of Wannier charge center (WCC) in the $k_z = 0$ plane. The insert in (f) shows the Brillouin zone of hexagonal cell and orthogonal cell. (g) Top view of a nanoribbon of β_2-$SrGa_2S_4$ with 20 unit cells. The edge is along (010) of the orthogonal cell. Brillouin zones of the orthogonal and hexagonal cells are shown in red solid-line square and rhombus, respectively.

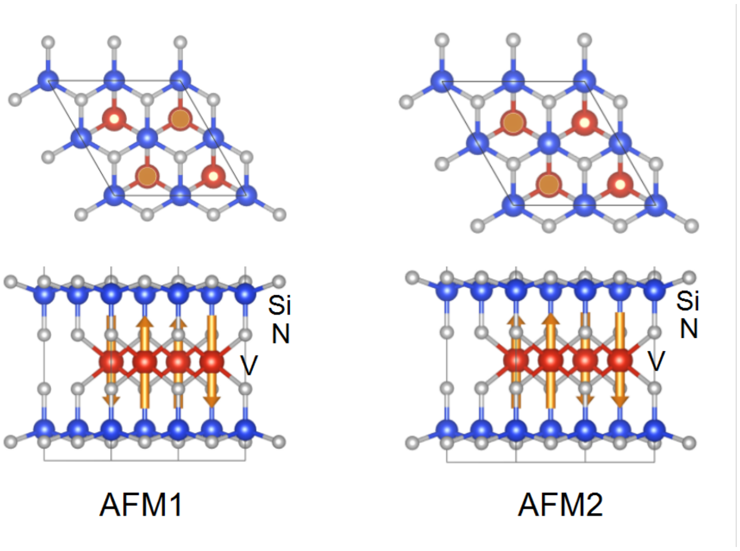

Supplementary Fig. 9. (color online) Antiferromagnetic configurations AFM1 and AMF2.

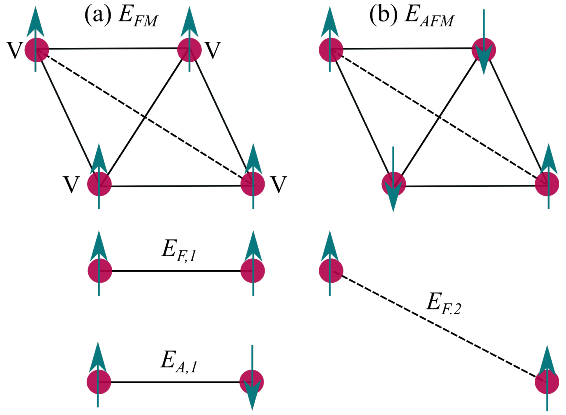

Supplementary Fig. 10. The magnetic configurations for the calculation of the magnetic exchange constant J of δ_4-VSi_2P_4.

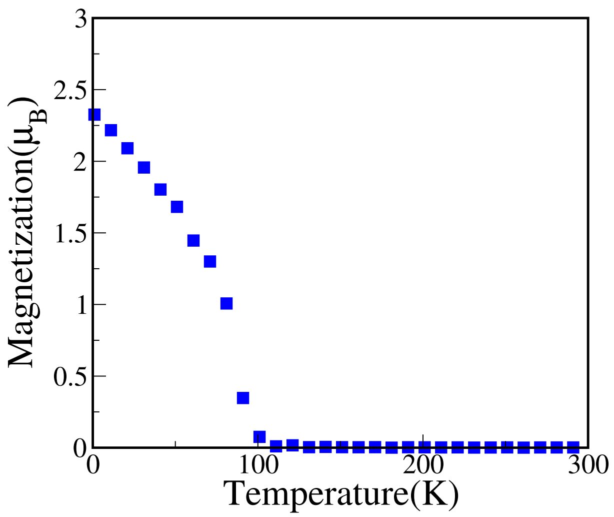

Supplementary Fig. 11. (color online) Monte Carlo (MC) simulations of the Heisenberg Hamiltonian, which uses a 100 × 100 2D lattice and 50000 Metropolis loops to achieve thermal equilibrium for each temperature step.

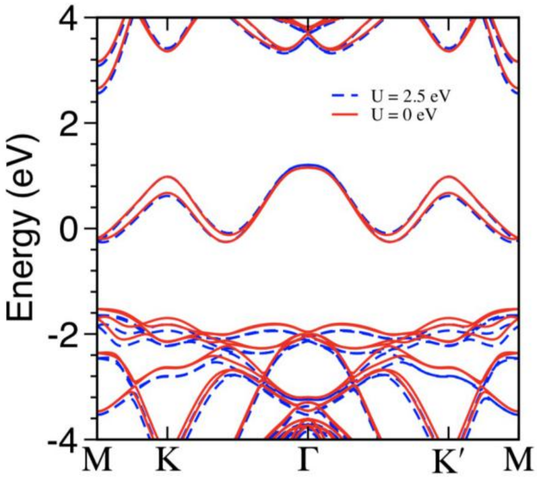

Supplementary Fig. 12. (color online) Electronic bands of α_1-$TaSi_2N_4$ with and without Hubbard U.

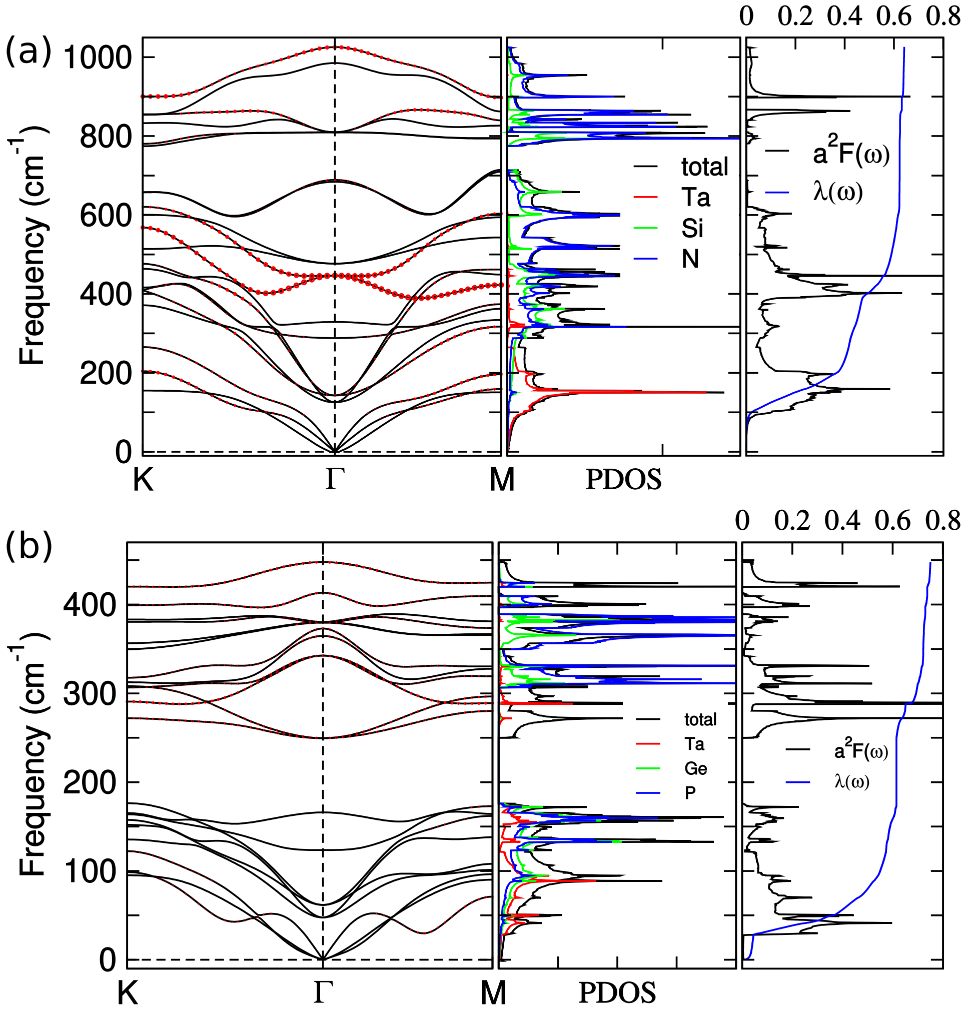

Supplementary Fig. 13. (color online) Phonon spectrum, phonon DOS, Eliashberg function $\alpha^2F(\omega)$ and EPC strength $\lambda(\omega)$ of α_1-$TaSi_2N_4$ (a) with the inclusion of SOC and α_2-$TaGe_2P_4$ (b) without the inclusion of SOC, where the size of the red circles represents the strength of phonon linewidth $\gamma_{\boldsymbol{q},\nu}$,

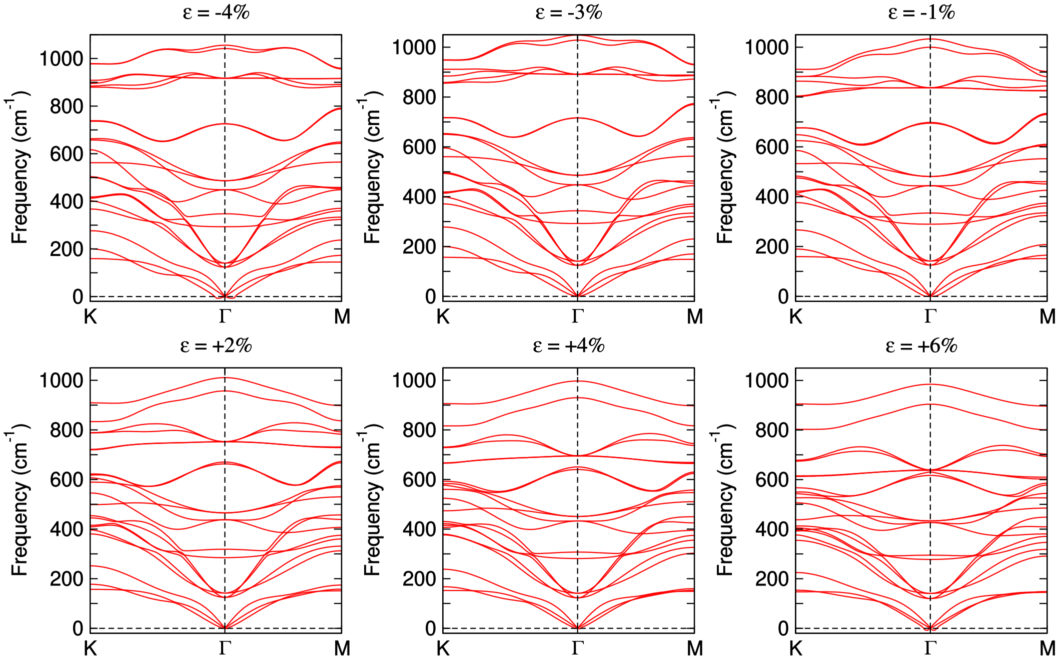

Supplementary Fig. 14. (color online) Phonon spectrum of α_1-$TaSi_2N_4$ monolayer with strain ranging from -4% to 6%.

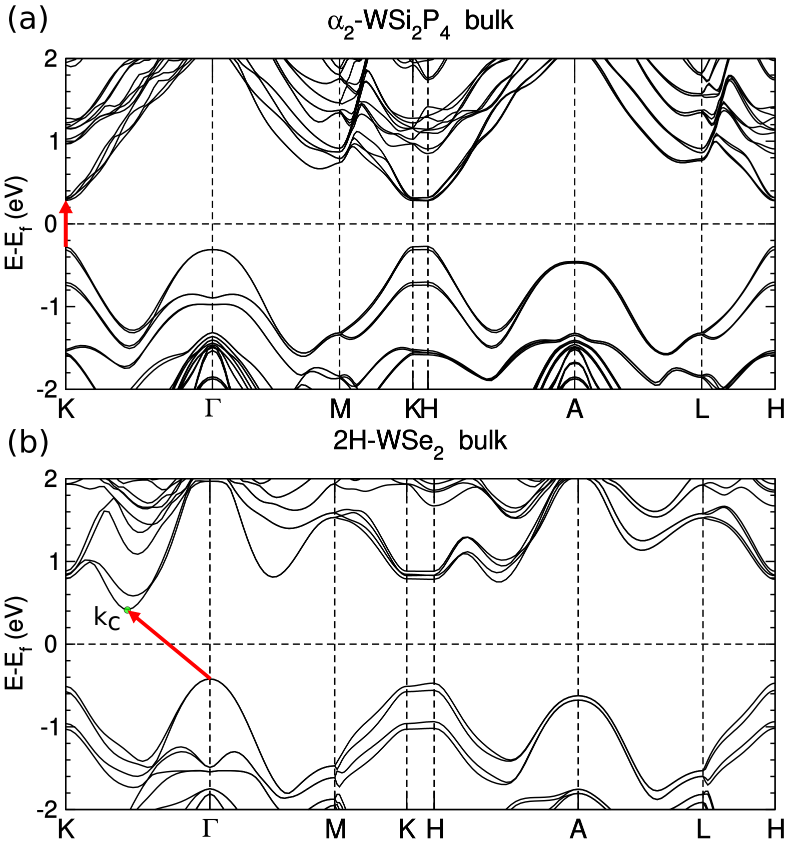

Supplementary Fig. 15. (color online) Electronic structures of bulk phase of α_2-WSi_2P_4 (a) and $2H$-WSe_2 (b) with AB stacking.

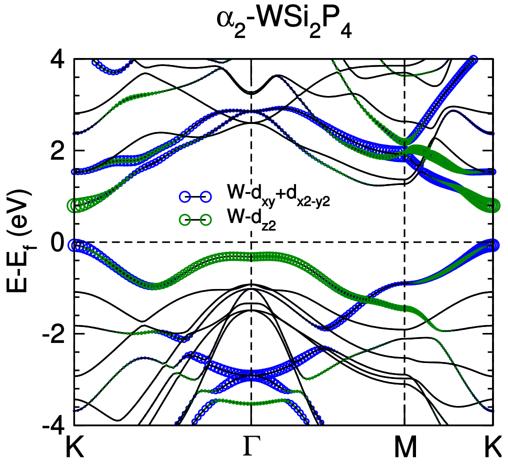

Supplementary Fig. 16. (color online) Orbital-resolved electronic structures of α_2-WSi_2P_4.

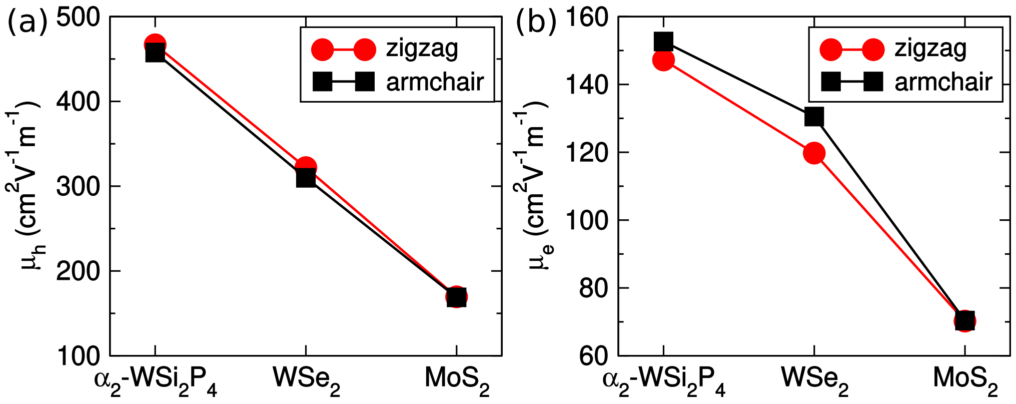

Supplementary Fig. 17. (color online) Carrier mobilities of α_2-WSi_2P_4, WSe_2 and MoS_2. The parameters used to calculate hole and electron mobility are listed in **Supplementary Table 8**

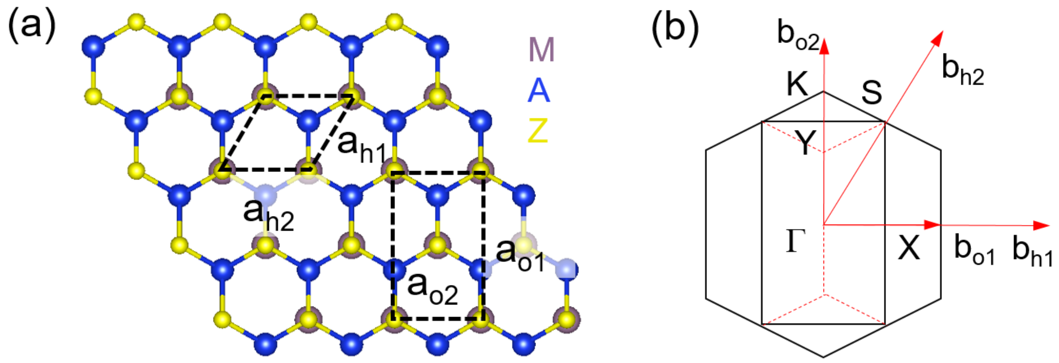

Supplementary Fig. 18. (color online) (a) The top view of α_2-MA_2Z_4 monolayer. Black dashed lines mark the hexagonal primitive cell and orthogonal supercell. (b) The first Brillouin zone of these two lattices. Red dash lines show the folding of first Brillouin zone of hexagonal cell into that of orthogonal cell.

Supplementary Table 1. Structural properties of 30 MA_2Z_4 monolayer candidates. SG denotes the space group. a is the in-plane lattice constant. xy represent the x and y coordinates of the atomic positions in the order of N1-N2-N3-N4-Mo1-Si1-Si2. a, b and c represent the sites, i.e., (0.000,0.000), (0.3333,0.6666) and (0.6666,0.3333), respectively. z_{N1}, z_{N2}, z_{N3}, z_{N4}, z_{Mo1}, z_{Si1} and z_{Si2} are the z component of the atomic positions. The coordinates are given in in fractional coordinates. The c axis of unit cell for computation is about 30 Å. An example of the structural file (POSCAR) for α_1-$MoSi_2N_4$ is given.

No.	phase	SG	a (Å)	xy	z_{N1}	z_{N2}	z_{N3}	z_{N4}	z_{Mo1}	z_{Si1}	z_{Si2}
1	α_1-$MoSi_2N_4$	P$\bar{6}$m2	2.909	baabcaa	0.000	0.075	0.158	0.233	0.117	0.017	0.217
2	α_2-$MoSi_2N_4$	P$\bar{6}$m2	2.901	caaccaa	0.000	0.076	0.160	0.236	0.118	0.017	0.218
3	α_3-$MoSi_2N_4$	P$\bar{6}$m2	2.835	aaaacbb	0.000	0.083	0.170	0.253	0.127	0.022	0.231
4	α_4-$MoSi_2N_4$	P$\bar{6}$m2	2.842	caaccbb	0.000	0.072	0.161	0.234	0.117	0.019	0.214
5	α_5-$MoSi_2N_4$	P$\bar{6}$m2	2.864	baabccc	0.000	0.070	0.157	0.228	0.114	0.019	0.209
6	α_6-$MoSi_2N_4$	P$\bar{6}$m2	2.845	aaaaccc	0.000	0.082	0.168	0.250	0.125	0.022	0.227
7	β_1-$MoSi_2N_4$	P$\bar{3}$m1	2.909	aacbabc	0.000	0.237	0.076	0.161	0.118	0.220	0.017
8	β_2-$MoSi_2N_4$	P$\bar{3}$m1	2.921	bccbabc	0.000	0.234	0.075	0.159	0.117	0.217	0.017
9	β_3-$MoSi_2N_4$	P$\bar{3}$m1	2.874	bccbaaa	0.000	0.226	0.069	0.157	0.113	0.207	0.019
10	β_4-$MoSi_2N_4$	P$\bar{3}$m1	2.857	cbcbaaa	0.000	0.249	0.081	0.167	0.124	0.226	0.022
11	β_5-$MoSi_2N_4$	P$\bar{3}$m1	2.842	ccabcab	0.000	0.235	0.072	0.163	0.118	0.216	0.020
12	β_6-$MoSi_2N_4$	P$\bar{3}$m1	2.841	cbcbacb	0.000	0.256	0.084	0.172	0.128	0.234	0.022
13	β_7-$MoSi_2N_4$	P3m1	2.900	abbcaab	0.000	0.230	0.076	0.162	0.119	0.211	0.017
14	β_8-$MoSi_2N_4$	P3m1	2.933	ccbcaab	0.000	0.236	0.075	0.154	0.118	0.216	0.017
15	β_9-$MoSi_2N_4$	P3m1	2.878	cabcabb	0.000	0.235	0.075	0.163	0.118	0.216	0.017
16	β_{10}-$MoSi_2N_4$	P3m1	2.853	babcaba	0.000	0.241	0.081	0.169	0.123	0.222	0.022
17	β_{11}-$MoSi_2N_4$	P3m1	2.876	acbcabb	0.000	0.247	0.076	0.163	0.120	0.225	0.018
18	β_{12}-$MoSi_2N_4$	P3m1	2.859	ccbcaba	0.000	0.241	0.069	0.157	0.113	0.219	0.019
19	γ_1-$MoSi_2N_4$	P3m1	2.882	babbcaa	0.000	0.075	0.161	0.246	0.118	0.017	0.224
20	γ_2-$MoSi_2N_4$	P3m1	2.872	cabccaa	0.000	0.076	0.165	0.236	0.120	0.018	0.217
21	γ_3-$MoSi_2N_4$	P3m1	2.868	babbccc	0.000	0.068	0.156	0.237	0.113	0.019	0.215
22	δ_1-$MoSi_2N_4$	P3m1	2.871	aaccabc	0.000	0.235	0.076	0.163	0.119	0.216	0.018
23	δ_2-$MoSi_2N_4$	P3m1	2.872	bcccabc	0.000	0.244	0.076	0.161	0.118	0.222	0.017
24	δ_3-$MoSi_2N_4$	P3m1	2.854	bcccaaa	0.000	0.239	0.070	0.157	0.114	0.217	0.019
25	δ_4-$MoSi_2N_4$	P3m1	2.905	abccacc	0.000	0.235	0.076	0.159	0.118	0.218	0.017
26	δ_5-$MoSi_2N_4$	P3m1	2.839	caccabb	0.000	0.243	0.083	0.171	0.126	0.224	0.022
27	δ_6-$MoSi_2N_4$	P3m1	2.888	bbccaac	0.000	0.230	0.075	0.160	0.117	0.212	0.017
28	δ_7-$MoSi_2N_4$	P3m1	2.854	baccaba	0.000	0.230	0.070	0.158	0.114	0.211	0.019
29	δ_8-$MoSi_2N_4$	P3m1	2.841	ccccaba	0.000	0.251	0.082	0.168	0.125	0.229	0.022
30	δ_9-$MoSi_2N_4$	P3m1	2.875	acccaac	0.000	0.243	0.076	0.161	0.119	0.221	0.018

Exampled by α_1-$MoSi_2N_4$.
POSCAR file:
α_1-$MoSi_2N_4$
1
a 0.00 0.00 # $a1$
-0.5a $\sqrt{3}a/2$ 0.00 # $a2$
0.00 0.00 30.0 # $a3$
N Mo Si
4 1 2
Direct
0.3333 0.6666 z_{N1} # b
0.0000 0.0000 z_{N2} # a
0.0000 0.0000 z_{N3} # a
0.3333 0.6666 z_{N4} # b
0.6666 0.3333 z_{Mo1} # c
0.0000 0.0000 z_{Si1} # a
0.0000 0.0000 z_{Si2} # a

Supplementary Table 2. Structural and electronic properties of MA_2Z_4 monolayer materials. a represents the lattice constant. Bond lengthes are given by d^v_{A-Z}, d_{Z-M} and d^h_{A-Z}, where v or h denotes the bond along vertical or horizontal direction. E_g^{PBE} and E_g^{HSE06} denote band gap calculated by PBE and HSE06 calculations, respectively. μ_B is the units of magnetic moment.

VEC	No.	Name	a (Å)	d^v_{A-Z} (Å)	d_{Z-M} (Å)	d^h_{A-Z} (Å)	E_g^{PBE} (eV)	E_g^{HSE06} (eV)	mag^{PBE} (μ_B)	mag^{HSE06} (μ_B)	Phase	Dynamics (Y/N)
32 VEC	01	$TiSi_2N_4$	2.95	1.77	2.04	1.75	0.61(Γ-M)	1.60(Γ-M)	—	—	β_2	N
	02	$ZrSi_2N_4$	3.05	1.83	2.16	1.75	1.00(Γ-M)	1.98(Γ-M)	—	—	β_2	Y
	03	$HfSi_2N_4$	3.04	1.82	2.14	1.75	1.21(Γ-M)	2.21(Γ-M)	—	—	β_2	Y
	04	$TiGe_2N_4$	3.08	1.89	2.08	1.89	0.82(Γ-M)	1.86(Γ-M)	—	—	β_2	N
	05	$ZrGe_2N_4$	3.19	1.93	2.19	1.89	1.04(Γ-Γ)	2.34(Γ-Γ)	—	—	β_2	Y
	06	$HfGe_2N_4$	3.18	1.93	2.17	1.89	1.15(Γ-Γ)	2.45(Γ-Γ)	—	—	β_2	Y
	07	$TiSi_2P_4$	3.53	2.27	2.49	2.22	—	—	—	—	β_2	Y
	08	$ZrSi_2P_4$	3.61	2.30	2.61	2.22	—	—	—	—	β_2	Y
	09	$HfSi_2P_4$	3.61	2.30	2.59	2.22	—	—	—	—	β_2	Y
	10	$TiGe_2P_4$	3.64	2.36	2.51	2.32	—	—	—	—	β_2	Y
	11	$ZrGe_2P_4$	3.72	2.38	2.63	2.32	—	—	—	—	β_2	Y
	12	$HfGe_2P_4$	3.72	2.38	2.60	2.33	—	—	—	—	β_2	Y
	13	$TiSi_2As_4$	3.70	2.40	2.59	2.34	—	—	—	—	β_2	Y
	14	$ZrSi_2As_4$	3.77	2.42	2.71	2.33	—	—	—	—	β_2	Y
	15	$HfSi_2As_4$	3.78	2.42	2.68	2.33	—	—	—	—	β_2	Y
	16	$TiGe_2As_4$	3.80	2.47	2.60	2.44	—	—	—	—	β_2	Y
	17	$ZrGe_2As_4$	3.87	2.50	2.72	2.43	—	—	—	—	β_2	Y
	18	$HfGe_2As_4$	3.88	2.50	2.70	2.43	—	—	—	—	β_2	Y
33 VEC	19	VSi_2N_4	2.88	1.75	2.03	1.75	—	—	0.97	1.00	α_1	Y
	20	$NbSi_2N_4$	2.96	1.78	2.13	1.75	—	—	0.57	1.00	α_1	Y
	21	$TaSi_2N_4$	2.97	1.78	2.13	1.75	—	—	—	—	α_1	Y
	22	VGe_2N_4	3.05	1.87	2.06	1.89	—	—	0.98	1.00	β_2	N
	23	$NbGe_2N_4$	3.09	1.89	2.16	1.90	—	—	0.72	1.00	α_1	Y
	24	$TaGe_2N_4$	3.08	1.87	2.15	1.88	—	—	0.49	1.00	α_1	Y
	25	VSi_2P_4	3.48	2.25	2.42	2.24	—	—	1.00	1.00	δ_4	Y
	26	$NbSi_2P_4$	3.53	2.27	2.52	2.23	—	—	—	—	α_1	Y
	27	$TaSi_2P_4$	3.54	2.27	2.52	2.24	—	—	—	—	α_1	Y
	28	VGe_2P_4	3.56	2.33	2.44	2.36	—	—	1.00	1.00	α_2	Y
	29	$NbGe_2P_4$	3.62	2.35	2.53	2.36	—	—	—	—	α_2	Y
	30	$TaGe_2P_4$	3.61	2.34	2.53	2.36	—	—	—	—	α_2	Y
	31	VSi_2As_4	3.64	2.38	2.55	2.38	—	—	1.00	1.00	α_2	Y
	32	$NbSi_2As_4$	3.68	2.39	2.63	2.37	—	—	—	—	α_2	Y
	33	$TaSi_2As_4$	3.68	2.39	2.62	2.38	—	—	—	—	α_2	Y
	34	VGe_2As_4	3.72	2.45	2.57	2.47	—	—	1.00	1.00	α_2	Y
	35	$NbGe_2As_4$	3.77	2.46	2.64	2.48	—	—	—	—	α_2	Y
	36	$TaGe_2As_4$	3.77	2.46	2.64	2.48	—	—	—	—	α_2	Y
34 VEC	37	$CrSi_2N_4$	2.84	1.73	2.00	1.75	0.49(Γ-K)	0.94(K-K)	—	—	α_1	Y
	38	$MoSi_2N_4$	2.91	1.75	2.09	1.75	1.74(Γ-K)	2.31(Γ-K)	—	—	α_1	Y
	39	WSi_2N_4	2.91	1.76	2.10	1.75	2.08(Γ-K)	2.66(Γ-K)	—	—	α_1	Y
	40	$CrGe_2N_4$	3.06	1.88	2.04	1.89	—	—	2.00	—	β_2	N
	41	$MoGe_2N_4$	3.02	1.85	2.12	1.87	0.99(ΓK-K)	1.38(ΓK-K)	—	—	α_1	Y
	42	WGe_2N_4	3.02	1.85	2.13	1.88	1.29(ΓK-K)	1.69(ΓK-K)	—	—	α_1	Y
	43	$CrSi_2P_4$	3.41	2.23	2.37	2.27	0.34(ΓK-K)	0.65(K-K)	—	—	α_2	Y
	44	$MoSi_2P_4$	3.46	2.25	2.46	2.26	0.91(K-K)	1.19(K-K)	—	—	α_2	Y
	45	WSi_2P_4	3.46	2.25	2.46	2.26	0.86(K-K)	1.11(K-K)	—	—	α_2	Y
	46	$CrGe_2P_4$	3.49	2.31	2.39	2.36	0.04(ΓK-K)	0.36(ΓK-K)	—	—	α_2	Y
	47	$MoGe_2P_4$	3.53	2.32	2.47	2.34	0.56(ΓK-K)	0.95(ΓK-K)	—	—	α_2	Y
	48	WGe_2P_4	3.54	2.32	2.47	2.35	0.63(ΓK-K)	0.89(K-K)	—	—	α_2	Y
	49	$CrSi_2As_4$	3.68	2.40	2.51	2.37	—	—	2.37	0.74	β_1	N
	50	$MoSi_2As_4$	3.61	2.37	2.57	2.37	0.74(K-K)	1.02(K-K)	—	—	α_2	Y
	51	WSi_2As_4	3.61	2.37	2.57	2.38	0.71(K-K)	0.95(K-K)	—	—	α_2	Y
	52	$CrGe_2As_4$	3.78	2.47	2.53	2.47	—	—	2.46	2.74	β_1	N
	53	$MoGe_2As_4$	3.69	2.44	2.58	2.47	0.47(ΓK-K)	0.83(ΓK-K)	—	—	α_2	Y
	54	WGe_2As_4	3.69	2.44	2.58	2.47	0.50(ΓK-ΓK)	0.78(K-K)	—	—	α_2	Y

Supplementary Table 3. Structural and electronic properties of MA_2Z_4 monolayers. a represents the lattice constant. Bond lengthes are given by d^v_{A-Z}, d_{Z-M} and d^h_{A-Z}, where v and h represent the bond along the vertical and horizontal directions, respectively. E_g^{PBE} and E_g^{HSE06} denote the band gap calculated by PBE and HSE06 calculations, respectively. Z_2^{PBE} and Z_2^{HSE06} are the topological Z_2 index derived by the PBE and HSE06 calculations, respectively.

No.	Name	a (Å)	d^v_{A-Z} (Å)	d_{Z-M} (Å)	d^h_{A-Z} (Å)	E_g^{PBE} (eV)	E_g^{HSE06} (eV)	Z_2^{PBE}	Z_2^{HSE06}	Phase	Dynamics (Y/N)
01	$MgAl_2S_4$	3.71	2.35	2.55	2.17	2.08	3.19	0	0	β_2	Y
02	$CaAl_2S_4$	3.80	2.38	2.74	2.16	1.65	2.75	0	0	β_2	Y
03	$SrAl_2S_4$	3.86	2.40	2.87	2.16	1.45	2.53	0	0	β_2	Y
04	$ZnAl_2S_4$	3.69	2.34	2.50	2.18	1.95	3.03	0	0	β_1	Y
05	$CdAl_2S_4$	3.78	2.37	2.65	2.17	1.46	2.51	0	0	β_1	Y
06	$HgAl_2S_4$	3.79	2.38	2.68	2.18	0.91	1.91	0	0	β_1	N
07	$MgGa_2S_4$	3.75	2.39	2.55	2.22	1.18	2.26	0	0	β_2	Y
08	$CaGa_2S_4$	3.84	2.42	2.74	2.21	0.69	1.74	0	0	β_2	Y
09	$SrGa_2S_4$	3.90	2.44	2.87	2.21	0.47	1.53	0	0	β_2	Y
10	$ZnGa_2S_4$	3.72	2.38	2.51	2.23	1.02	2.12	0	0	β_2	Y
11	$CdGa_2S_4$	3.82	2.41	2.65	2.22	0.56	1.56	0	0	β_1	Y
12	$HgGa_2S_4$	3.83	2.42	2.68	2.23	0.07	0.98	0	0	β_1	N
13	$MgAl_2Se_4$	3.92	2.50	2.68	2.31	1.07	2.14	0	0	β_2	Y
14	$CaAl_2Se_4$	4.00	2.53	2.86	2.30	0.69	1.76	0	0	β_2	Y
15	$SrAl_2Se_4$	4.06	2.55	2.99	2.30	0.53	1.59	0	0	β_2	Y
16	$ZnAl_2Se_4$	3.89	2.50	2.63	2.32	1.02	2.10	0	0	β_1	Y
17	$CdAl_2Se_4$	3.98	2.52	2.77	2.32	0.58	1.59	0	0	β_1	Y
18	$HgAl_2Se_4$	3.99	2.53	2.80	2.32	0.12	1.01	0	0	β_1	N
19	$MgGa_2Se_4$	3.95	2.53	2.68	2.35	0.36	1.35	0	0	β_2	Y
20	$CaGa_2Se_4$	4.04	2.56	2.86	2.34	0.04	0.89	1	0	β_2	Y
21	$SrGa_2Se_4$	4.10	2.58	2.99	2.33	0.08	0.72	1	0	β_2	Y
22	$ZnGa_2Se_4$	3.92	2.52	2.63	2.36	0.31	1.30	0	0	β_2	N
23	$CdGa_2Se_4$	4.02	2.56	2.77	2.36	0.02	0.76	0	0	β_2	N
24	$HgGa_2Se_4$	4.04	2.56	2.80	2.37	—	0.17	0	0	β_2	N
25	$MgAl_2Te_4$	4.27	2.74	2.91	2.55	0.5	1.40	0	0	β_2	Y
26	$CaAl_2Te_4$	4.33	2.76	3.09	2.53	0.09	1.01	0	0	β_2	Y
27	$SrAl_2Te_4$	4.38	2.77	3.22	2.52	0	0.88	1	0	β_2	Y
28	$ZnAl_2Te_4$	4.23	2.72	2.84	2.56	0.63	1.54	0	0	β_1	N
29	$CdAl_2Te_4$	4.31	2.75	2.97	2.55	0.17	1.09	0	0	β_1	N
30	$HgAl_2Te_4$	4.33	2.76	2.99	2.56	—	0.65	0	0	β_1	N
31	$MgGa_2Te_4$	4.29	2.75	2.90	2.57	0.06	0.68	1	0	β_2	Y
32	$CaGa_2Te_4$	4.36	2.77	3.08	2.55	—	0.20	1	1	β_2	Y
33	$SrGa_2Te_4$	4.41	2.79	3.22	2.55	—	0.05	1	1	β_2	Y
34	$ZnGa_2Te_4$	4.24	2.74	2.84	2.59	0.01	0.79	0	0	β_2	N
35	$CdGa_2Te_4$	4.34	2.77	2.96	2.58	—	0.35	0	0	β_2	N
36	$HgGa_2Te_4$	4.35	2.77	2.99	2.60	—	—	0	0	β_1	N

Supplementary Table 4. Enthalpies of formation E_f (eV/atom) of α_{1-6}- and β_{1-12}-MA_2Z_4 monolayer materials, respectively, where M = Ti, Zr, Hf, V, Nb, Ta, Cr, Mo, W; A = Si and Ge; Z = N, P and As.

No.	Name	α_1	α_2	α_3	α_4	α_5	α_6	β_1	β_2	β_3	β_4	β_5	β_6	β_7	β_8	β_9	β_{10}	β_{11}	β_{12}
01	$TiSi_2N_4$	-1.052	-1.036	-0.262	-0.633	-0.668	-0.282	-1.091	**-1.119**	-0.765	-0.405	-0.708	-0.369	-0.920	-0.756	-0.911	-0.555	-0.722	-0.567
02	$ZrSi_2N_4$	-0.991	-0.986	-0.183	-0.558	-0.570	-0.196	-1.013	**-1.028**	-0.631	-0.272	-0.595	-0.235	-0.809	-0.647	-0.811	-0.433	-0.618	-0.434
03	$HfSi_2N_4$	-1.053	-1.047	-0.217	-0.602	-0.617	-0.231	-1.091	**-1.105**	-0.694	-0.333	-0.661	-0.298	-0.881	-0.714	-0.882	-0.496	-0.686	-0.496
04	$TiGe_2N_4$	-0.331	-0.326	0.242	-0.009	-0.021	0.087	-0.395	**-0.409**	-0.142	0.083	-0.105	0.115	-0.263	-0.154	-0.255	-0.008	-0.132	-0.014
05	$ZrGe_2N_4$	-0.378	-0.376	0.220	-0.040	-0.039	0.221	-0.426	**-0.433**	-0.133	0.098	-0.113	0.128	-0.272	-0.161	-0.272	-0.006	-0.142	-0.003
06	$HfGe_2N_4$	-0.425	-0.423	0.193	-0.073	-0.074	0.195	-0.489	**-0.496**	-0.183	0.051	-0.165	0.077	-0.328	-0.215	-0.329	-0.056	-0.197	-0.053
07	$TiSi_2P_4$	-0.327	-0.316	0.171	-0.034	-0.204	-0.101	-0.335	**-0.348**	-0.193	-0.010	-0.073	0.075	-0.270	-0.154	-0.204	-0.026	-0.128	-0.052
08	$ZrSi_2P_4$	-0.366	-0.349	0.080	-0.077	-0.206	-0.129	-0.372	**-0.390**	-0.252	-0.074	-0.114	0.043	-0.326	-0.206	-0.245	-0.074	-0.174	-0.097
09	$HfSi_2P_4$	-0.357	-0.342	0.122	-0.060	-0.155	-0.095	-0.366	**-0.383**	-0.231	-0.048	-0.104	0.049	-0.311	-0.190	-0.238	-0.059	-0.169	-0.085
10	$TiGe_2P_4$	-0.194	-0.190	0.153	0.020	-0.117	-0.018	-0.196	**-0.200**	-0.011	0.004	0.051	0.123	-0.113	-0.040	-0.071	0.071	-0.026	0.054
11	$ZrGe_2P_4$	-0.253	-0.244	0.023	0.017	-0.127	-0.132	-0.253	**-0.262**	-0.096	-0.096	-0.013	0.077	-0.190	-0.116	-0.135	0.007	-0.097	-0.008
12	$HfGe_2P_4$	-0.243	-0.235	0.066	0.026	-0.106	-0.091	-0.249	**-0.258**	-0.076	-0.070	-0.006	0.083	-0.178	-0.102	-0.131	0.018	-0.091	0.004
13	$TiSi_2As_4$	-0.212	-0.207	0.079	0.019	-0.098	-0.013	-0.225	**-0.231**	-0.081	0.059	-0.011	0.116	-0.161	-0.065	-0.109	0.043	-0.054	0.020
14	$ZrSi_2As_4$	-0.266	-0.255	0.070	-0.037	-0.116	-0.045	-0.275	**-0.284**	-0.157	0.002	-0.063	0.066	-0.232	-0.133	-0.168	-0.024	-0.119	-0.044
15	$HfSi_2As_4$	-0.240	-0.231	0.090	-0.007	-0.081	-0.011	-0.253	**-0.262**	-0.119	0.038	-0.036	0.089	-0.201	-0.102	-0.143	0.006	-0.097	-0.014
16	$TiGe_2As_4$	-0.163	-0.162	0.020	0.014	-0.082	-0.053	-0.172	**-0.172**	-0.011	0.008	0.016	0.086	-0.097	-0.039	-0.065	0.045	-0.032	0.035
17	$ZrGe_2As_4$	-0.231	-0.225	0.084	-0.017	-0.131	-0.111	-0.236	**-0.239**	-0.093	-0.076	-0.046	0.033	-0.180	-0.121	-0.140	-0.025	-0.113	0.030
18	$HfGe_2As_4$	-0.205	-0.200	0.140	0.007	-0.092	-0.072	-0.217	**-0.220**	-0.059	-0.038	-0.021	0.055	-0.153	-0.092	-0.118	0.003	-0.091	-0.003
19	VSi_2N_4	**-0.954**	-0.933	-0.196	-0.545	-0.619	-0.231	-0.913	-0.949	-0.637	-0.252	-0.540	-0.211	-0.776	-0.600	-0.739	-0.401	-0.558	-0.421
20	$NbSi_2N_4$	**-0.989**	-0.964	-0.206	-0.552	-0.628	-0.245	-0.927	-0.966	-0.629	-0.243	-0.534	-0.206	-0.581	-0.595	-0.745	-0.361	-0.584	-0.415
21	$TaSi_2N_4$	**-1.009**	-0.978	-0.205	-0.551	-0.634	-0.244	-0.944	-0.984	-0.633	-0.252	-0.537	-0.222	-0.627	-0.640	-0.754	-0.399	-0.629	-0.429
22	VGe_2N_4	-0.173	-0.167	0.359	0.138	0.098	0.320	-0.171	**-0.186**	0.057	0.280	0.123	0.312	-0.056	0.017	-0.028	0.164	0.061	0.182
23	$NbGe_2N_4$	**-0.293**	-0.280	0.254	0.036	-0.016	0.222	-0.255	-0.275	-0.029	0.173	0.042	0.186	-0.152	-0.107	-0.111	0.065	-0.090	0.057
24	$TaGe_2N_4$	**-0.310**	-0.295	0.238	0.031	-0.027	0.214	-0.276	-0.298	-0.043	0.134	0.028	0.144	-0.176	-0.144	-0.144	0.031	-0.139	0.016
25	VSi_2P_4	-0.2458	-0.244	0.112	0.026	-0.111	0.023	-0.221	-0.225	-0.061	0.127	0.009	0.157	-0.149	-0.045	-0.099	0.079	-0.041	0.053
26	$NbSi_2P_4$	**-0.321**	-0.317	0.210	-0.049	-0.156	0.005	-0.290	-0.294	-0.138	0.087	-0.065	0.081	-0.231	-0.117	-0.180	-0.001	-0.122	-0.029
27	$TaSi_2P_4$	**-0.297**	-0.293	0.256	-0.025	-0.113	0.050	-0.270	-0.270	-0.101	0.123	-0.048	0.095	-0.205	-0.098	-0.163	0.021	-0.109	-0.007
28	VGe_2P_4	-0.091	**-0.098**	0.130	0.117	0.011	0.059	-0.069	-0.063	0.114	0.142	0.145	0.228	0.025	0.065	0.045	0.178	0.072	0.181
29	$NbGe_2P_4$	-0.176	**-0.183**	0.211	0.060	-0.028	0.019	-0.151	-0.139	0.053	0.105	0.053	0.142	-0.065	-0.020	-0.053	0.088	-0.025	0.094
30	$TaGe_2P_4$	-0.153	**-0.161**	0.258	0.081	0.017	0.069	-0.131	-0.122	0.078	0.140	0.064	0.153	-0.043	-0.010	-0.042	0.104	-0.015	0.111
31	VSi_2As_4	-0.101	**-0.104**	0.201	0.115	0.035	0.145	-0.090	-0.088	0.065	0.176	0.100	0.228	-0.013	0.066	0.022	0.169	0.064	0.154
32	$NbSi_2As_4$	-0.166	**-0.170**	0.163	0.036	-0.022	0.085	-0.145	-0.141	0.004	0.164	0.018	0.139	-0.087	-0.001	-0.056	0.092	-0.014	0.079
33	$TaGe_2As_4$	-0.122	**-0.127**	0.207	0.083	0.036	0.143	-0.106	-0.098	0.063	0.217	0.063	0.176	-0.041	0.030	-0.020	0.131	0.017	0.119
34	VGe_2As_4	-0.035	**-0.043**	0.159	0.139	0.077	0.110	-0.028	-0.022	0.120	0.137	0.124	0.217	0.054	0.085	0.071	0.163	0.091	0.165
35	$NbGe_2As_4$	-0.106	**-0.119**	0.123	0.064	0.012	0.033	-0.090	-0.077	0.088	0.110	0.043	0.093	-0.017	0.011	-0.014	0.098	0.006	0.116
36	$TaGe_2As_4$	-0.065	**-0.078**	0.172	0.105	0.070	0.092	-0.056	-0.041	0.133	0.162	0.085	0.128	0.022	0.039	0.016	0.132	0.034	0.151
37	$CrSi_2N_4$	**-0.831**	-0.807	-0.082	-0.429	-0.512	-0.119	-0.731	-0.762	-0.458	-0.037	0.106	-0.014	-0.581	-0.401	-0.550	-0.195	-0.364	-0.225
38	$MoSi_2N_4$	**-0.955**	-0.931	-0.157	-0.517	-0.591	-0.189	-0.775	-0.803	-0.470	-0.057	-0.388	-0.052	-0.624	-0.433	-0.588	-0.225	-0.412	-0.263
39	WSi_2N_4	**-0.955**	-0.929	-0.147	-0.502	-0.579	-0.177	-0.757	-0.782	-0.434	-0.033	-0.363	-0.042	-0.599	-0.426	-0.565	-0.200	-0.401	-0.243
40	$CrGe_2N_4$	0.004	0.009	0.514	0.299	0.242	0.479	-0.001	**-0.014**	0.263	0.479	0.308	0.505	0.127	0.204	0.145	0.343	0.258	0.377
41	$MoGe_2N_4$	**-0.185**	-0.177	0.355	0.136	0.082	0.327	-0.044	-0.056	0.187	0.410	0.249	0.427	0.054	0.119	0.104	0.283	0.186	0.305
42	WGe_2N_4	**-0.187**	-0.178	0.352	0.144	0.088	0.327	-0.030	-0.042	0.205	0.393	0.266	0.408	0.056	0.108	0.118	0.281	0.179	0.304
43	$CrSi_2P_4$	-0.176	**-0.184**	0.186	0.082	-0.022	0.145	-0.122	-0.149	0.022	0.145	0.092	0.255	-0.051	0.008	-0.006	0.213	0.068	0.147
44	$MoSi_2P_4$	-0.294	**-0.303**	0.137	-0.030	-0.050	0.141	-0.210	-0.202	0.003	0.183	-0.003	0.141	-0.111	0.010	-0.100	0.107	-0.030	0.080
45	WSi_2P_4	-0.241	**-0.252**	0.185	0.021	0.022	0.210	-0.159	-0.146	0.070	0.259	0.051	0.192	-0.056	0.061	-0.048	0.161	0.018	0.135
46	$CrGe_2P_4$	0.001	**-0.015**	0.219	0.197	0.118	0.187	-0.006	-0.006	0.152	0.194	0.230	0.305	0.091	0.116	0.130	0.297	0.163	0.314
47	$MoGe_2P_4$	-0.127	**-0.144**	0.348	0.105	0.087	0.163	-0.052	-0.036	0.197	0.197	0.126	0.203	0.063	0.113	0.056	0.205	0.101	0.228
48	WGe_2P_4	-0.077	**-0.094**	0.423	0.152	0.168	0.238	-0.005	0.013	0.259	0.276	0.175	0.249	0.112	0.162	0.102	0.252	0.144	0.274
49	$CrSi_2As_4$	0.000	-0.013	0.266	0.195	0.122	0.244	**-0.0408**	-0.0407	0.118	0.200	0.186	0.300	0.059	0.105	0.083	0.213	0.151	0.215
50	$MoSi_2As_4$	-0.106	**-0.122**	0.215	0.091	0.095	0.230	-0.047	-0.034	0.142	0.253	0.105	0.198	0.044	0.136	0.046	0.193	0.098	0.219
51	WSi_2As_4	-0.025	**-0.042**	0.289	0.168	0.196	0.329	0.033	0.050	0.236	0.350	0.187	0.271	0.126	0.216	0.126	0.270	0.173	0.293
52	$CrGe_2As_4$	0.049	0.038	0.233	0.209	0.164	0.207	**0.009**	0.013	0.151	0.153	0.212	0.275	0.106	0.126	0.129	0.253	0.162	0.253
53	$MoGe_2As_4$	-0.030	**-0.051**	0.221	0.130	0.142	0.177	0.020	0.038	0.210	0.202	0.130	0.157	0.159	0.148	0.108	0.170	0.138	0.172
54	WGe_2As_4	0.048	**0.027**	0.304	0.204	0.244	0.274	0.095	0.117	0.306	0.303	0.208	0.228	0.194	0.228	0.183	0.242	0.208	0.245

Supplementary Table 5. Enthalpies of formation E_f (eV/atom) of γ_{1-3}- and δ_{1-9}-MA_2Z_4 monolayer materials, where M = Ti, Zr, Hf, V, Nb, Ta, Cr, Mo, W; A = Si and Ge; Z = N, P and As.

No.	Name	γ_1	γ_2	γ_3	δ_1	δ_2	δ_3	δ_4	δ_5	δ_6	δ_7	δ_8	δ_9
01	$TiSi_2N_4$	-0.738	-0.896	-0.586	-0.832	-0.654	-0.482	-1.044	-0.453	-0.855	-0.650	-0.272	-0.658
02	$ZrSi_2N_4$	-0.629	-0.800	-0.453	-0.768	-0.584	-0.388	-0.988	-0.375	-0.773	-0.564	-0.189	-0.586
03	$HfSi_2N_4$	-0.697	-0.872	-0.515	-0.821	-0.632	-0.429	-1.050	-0.415	-0.828	-0.609	-0.224	-0.636
04	$TiGe_2N_4$	-0.140	-0.247	-0.029	-0.165	-0.041	0.102	-0.329	0.168	-0.174	-0.015	0.149	-0.047
05	$ZrGe_2N_4$	-0.148	-0.267	-0.018	-0.206	-0.075	0.087	-0.377	0.088	-0.204	-0.040	0.221	-0.077
06	$HfGe_2N_4$	-0.203	-0.324	-0.067	-0.245	-0.112	0.056	-0.424	0.058	-0.246	-0.074	0.194	-0.116
07	$TiSi_2P_4$	-0.128	-0.199	-0.079	-0.168	-0.055	-0.165	-0.323	0.024	-0.227	-0.095	0.045	-0.185
08	$ZrSi_2P_4$	-0.176	-0.241	-0.132	-0.210	-0.117	-0.156	-0.359	0.003	-0.283	-0.143	-0.039	-0.224
09	$HfSi_2P_4$	-0.170	-0.232	-0.109	-0.196	-0.086	-0.130	-0.351	0.018	-0.264	-0.123	0.000	-0.200
10	$TiGe_2P_4$	-0.024	-0.069	0.034	-0.049	0.032	-0.100	-0.193	0.023	-0.084	-0.034	0.066	-0.083
11	$ZrGe_2P_4$	-0.042	-0.135	-0.038	-0.105	-0.062	-0.121	-0.250	0.019	-0.151	-0.023	-0.059	-0.155
12	$HfGe_2P_4$	-0.028	-0.128	-0.018	-0.092	-0.033	-0.099	-0.240	0.029	-0.133	-0.020	-0.018	-0.127
13	$TiSi_2As_4$	-0.052	-0.109	0.002	-0.075	0.000	-0.063	-0.211	0.071	-0.118	-0.010	0.041	-0.071
14	$ZrSi_2As_4$	-0.117	-0.169	-0.065	-0.130	-0.061	-0.092	-0.262	0.034	-0.189	-0.068	0.013	-0.126
15	$HfSi_2As_4$	-0.096	-0.143	-0.026	-0.101	-0.034	-0.057	-0.237	0.061	-0.154	-0.031	0.045	-0.085
16	$TiGe_2As_4$	-0.028	-0.067	0.010	-0.042	-0.009	-0.072	-0.164	0.018	-0.066	-0.019	-0.006	-0.057
17	$ZrGe_2As_4$	-0.109	-0.143	-0.048	-0.106	-0.035	-0.129	-0.230	-0.015	-0.141	-0.058	-0.020	-0.138
18	$HfGe_2As_4$	0.001	-0.120	-0.017	-0.079	0.007	-0.089	-0.204	0.010	-0.108	-0.024	0.035	-0.093
19	VSi_2N_4	-0.572	-0.722	-0.445	-0.735	-0.569	-0.428	-0.943	-0.372	-0.783	-0.579	-0.214	-0.586
20	$NbSi_2N_4$	-0.597	-0.728	-0.435	-0.755	-0.595	-0.437	-0.976	-0.380	-0.810	-0.590	-0.227	-0.614
21	$TaSi_2N_4$	-0.641	-0.737	-0.441	-0.762	-0.606	-0.440	-0.993	-0.381	-0.824	-0.594	-0.226	-0.627
22	VGe_2N_4	0.057	-0.020	0.149	-0.011	0.097	0.193	-0.170	0.240	-0.039	0.114	0.329	0.062
23	$NbGe_2N_4$	-0.095	-0.091	0.052	-0.120	-0.031	0.097	-0.287	0.136	-0.161	0.007	0.235	-0.064
24	$TaGe_2N_4$	-0.143	-0.139	0.019	-0.129	-0.055	0.084	-0.302	0.119	-0.177	-0.002	0.223	-0.082
25	VSi_2P_4	-0.038	-0.102	0.032	-0.102	-0.043	-0.057	**-0.2461**	0.091	-0.168	-0.037	0.040	-0.090
26	$NbSi_2P_4$	-0.120	-0.184	-0.017	-0.182	-0.035	-0.086	-0.320	0.021	-0.254	-0.108	0.090	-0.154
27	$TaSi_2P_4$	-0.107	-0.167	0.008	-0.161	-0.103	-0.045	-0.295	0.045	-0.223	-0.073	0.132	-0.117
28	VGe_2P_4	0.078	0.039	0.140	0.025	0.059	0.029	-0.095	0.124	-0.015	0.049	0.226	0.009
29	$NbGe_2P_4$	-0.019	-0.059	0.099	-0.068	0.070	-0.023	-0.180	0.096	-0.099	0.010	0.096	-0.071
30	$TaGe_2P_4$	-0.009	-0.048	0.113	-0.049	0.112	0.020	-0.157	0.121	-0.066	0.049	0.142	-0.038
31	VSi_2As_4	0.070	0.016	0.125	0.020	0.074	0.082	-0.104	0.179	-0.016	0.089	0.156	0.043
32	$NbSi_2As_4$	-0.008	-0.063	0.092	-0.060	-0.007	0.024	-0.169	0.104	-0.103	0.018	0.104	-0.033
33	$TaGe_2As_4$	0.023	-0.028	0.130	-0.020	0.031	0.084	-0.125	0.148	-0.052	0.071	0.153	0.017
34	VGe_2As_4	0.100	0.063	0.137	0.058	0.096	0.091	-0.041	0.153	0.045	0.102	0.128	0.060
35	$NbGe_2As_4$	0.014	-0.023	0.105	-0.027	0.006	0.013	-0.113	0.108	-0.041	0.043	0.060	-0.026
36	$TaGe_2As_4$	0.043	0.007	0.147	0.010	0.045	0.073	-0.072	0.149	0.010	0.095	0.110	0.023
37	$CrSi_2N_4$	-0.380	-0.537	-0.242	-0.615	-0.448	-0.315	-0.819	-0.256	-0.670	-0.473	-0.102	-0.459
38	$MoSi_2N_4$	-0.423	-0.578	-0.263	-0.720	-0.550	-0.390	-0.943	-0.338	-0.772	-0.558	-0.175	-0.558
39	WSi_2N_4	-0.411	-0.556	-0.232	-0.712	-0.546	-0.377	-0.942	-0.325	-0.766	-0.544	-0.164	-0.552
40	$CrGe_2N_4$	0.252	0.152	0.329	0.156	0.268	0.362	0.006	0.408	0.121	0.268	0.497	0.244
41	$MoGe_2N_4$	0.182	0.108	0.267	-0.018	0.092	0.207	-0.181	0.246	-0.054	0.105	0.342	0.074
42	WGe_2N_4	0.174	0.121	0.267	-0.014	0.089	0.210	-0.183	0.248	-0.052	0.112	0.342	0.073
43	$CrSi_2P_4$	0.069	-0.005	0.072	-0.036	0.036	0.052	-0.180	0.160	-0.086	0.043	0.114	0.004
44	$MoSi_2P_4$	-0.024	-0.106	0.123	-0.156	-0.068	0.040	-0.299	0.053	-0.178	-0.038	0.105	-0.016
45	WSi_2P_4	0.025	-0.055	0.164	-0.107	0.145	0.113	-0.247	0.102	-0.120	0.019	0.169	0.047
46	$CrGe_2P_4$	0.168	0.131	0.171	0.105	0.143	0.149	-0.008	0.208	0.079	0.140	0.173	0.105
47	$MoGe_2P_4$	0.111	0.047	0.229	-0.008	0.046	0.104	-0.136	0.160	-0.011	0.101	0.127	0.021
48	WGe_2P_4	0.153	0.092	0.297	0.039	0.097	0.179	-0.086	0.206	0.046	0.172	0.190	0.081
49	$CrSi_2As_4$	0.153	0.084	0.160	0.099	0.164	0.186	-0.007	0.244	0.084	0.179	0.255	0.147
50	$MoSi_2As_4$	0.108	0.037	0.221	0.000	0.078	0.160	-0.114	0.154	-0.003	0.111	0.225	0.073
51	WSi_2As_4	0.183	0.116	0.318	0.075	0.153	0.260	-0.034	0.229	0.083	0.197	0.311	0.152
52	$CrGe_2As_4$	0.167	0.129	0.172	0.137	0.183	0.186	0.043	0.221	0.152	0.197	0.221	0.162
53	$MoGe_2As_4$	0.150	0.096	0.209	0.057	0.109	0.155	-0.041	0.159	0.070	0.156	0.187	0.084
54	WGe_2As_4	0.220	0.171	0.301	0.131	0.187	0.254	0.037	0.235	0.153	0.246	0.272	0.167

Supplementary Table 6. Enthalpies of formation E_f (eV/atom) of α_{1-6}- and β_{1-12}-MA_2Z_4 monolayer materials, where M = Mg, Ca, Sr, Zn, Cd, Hg; A = Al and Ga; Z = S, Se and Te.

No.	Name	α_1	α_2	α_3	α_4	α_5	α_6	β_1	β_2	β_3	β_4	β_5	β_6	β_7	β_8	β_9	β_{10}	β_{11}	β_{12}
01	$MgAl_2S_4$	-1.102	-1.101	-1.101	-0.856	-1.094	-0.853	-1.1525	**-1.1542**	-0.782	-1.037	-1.042	-0.784	-1.117	-0.985	-1.114	-0.973	-0.982	-0.976
02	$CaAl_2S_4$	-1.278	-1.275	-1.250	-0.998	-1.240	-0.989	-1.2891	**-1.2931**	-0.512	-1.219	-1.227	-0.530	-1.258	-1.125	-1.258	-1.117	-1.119	-1.119
03	$SrAl_2S_4$	-1.229	-1.226	-1.183	-0.923	-1.176	-0.914	-1.2290	**-1.2329**	-0.448	-1.170	-1.176	-0.919	-1.194	-1.058	-1.196	-1.047	-1.052	-1.048
04	$ZnAl_2S_4$	-0.820	-0.821	-0.827	-0.591	-0.831	-0.604	**-0.8854**	-0.8843	-0.519	-0.768	-0.763	-0.506	-0.851	-0.719	-0.850	-0.710	-0.723	-0.714
05	$CdAl_2S_4$	-0.810	-0.812	-0.792	-0.559	-0.805	-0.573	**-0.8398**	-0.8375	-0.523	-0.774	-0.763	-0.511	-0.805	-0.672	-0.809	-0.680	-0.679	-0.681
06	$HgAl_2S_4$	-0.623	-0.628	-0.614	-0.417	-0.635	-0.431	**-0.6474**	-0.6429	-0.397	-0.615	-0.593	-0.387	-0.620	-0.493	-0.627	-0.521	-0.505	-0.519
07	$MgGa_2S_4$	-0.804	-0.803	-0.719	-0.513	-0.715	-0.514	-0.8487	**-0.8504**	-0.439	-0.656	-0.657	-0.438	-0.777	-0.665	-0.776	-0.613	-0.663	-0.616
08	$CaGa_2S_4$	-0.974	-0.972	-0.881	-0.431	-0.867	-0.653	-0.9827	**-0.9852**	-0.494	-0.845	-0.855	-0.491	-0.923	-0.810	-0.920	-0.765	-0.802	-0.766
09	$SrGa_2S_4$	-0.925	-0.923	-0.823	-0.447	-0.812	-0.588	-0.9248	**-0.9273**	-0.475	-0.804	-0.814	-0.439	-0.865	-0.749	-0.864	-0.705	-0.742	-0.705
10	$ZnGa_2S_4$	-0.526	-0.526	-0.449	-0.256	-0.459	-0.278	-0.5863	**-0.5869**	-0.195	-0.393	-0.379	-0.174	-0.514	-0.405	-0.519	-0.358	-0.412	-0.363
11	$CdGa_2S_4$	-0.513	-0.514	-0.421	-0.224	-0.439	-0.247	**-0.5400**	-0.5398	-0.194	-0.405	-0.387	0.032	-0.472	-0.361	-0.481	-0.331	-0.370	-0.333
12	$HgGa_2S_4$	-0.330	-0.331	-0.249	0.042	-0.276	-0.106	**-0.3521**	-0.3510	-0.064	-0.251	-0.221	0.207	-0.292	-0.187	-0.306	-0.176	-0.203	-0.176
13	$MgAl_2Se_4$	-0.907	-0.906	-0.899	-0.674	-0.888	-0.673	-0.9531	**-0.9546**	-0.602	-0.835	-0.841	-0.602	-0.918	-0.796	-0.913	-0.779	-0.793	-0.785
14	$CaAl_2Se_4$	-1.103	-1.100	-1.083	-0.852	-1.065	-0.838	-1.1113	**-1.1152**	-0.493	-1.042	-1.057	-0.520	-1.087	-0.966	-1.082	-0.957	-0.958	-0.960
15	$SrAl_2Se_4$	-1.073	-1.069	-1.044	-0.807	-1.028	-0.793	-1.0720	**-1.0762**	-0.463	-1.018	-1.032	-0.458	-1.048	-0.924	-1.045	-0.915	-0.916	-0.918
16	$ZnAl_2Se_4$	-0.653	-0.654	-0.655	-0.445	-0.655	-0.460	**-0.7164**	-0.7157	-0.386	-0.595	-0.588	-0.373	-0.682	-0.562	-0.680	-0.549	-0.566	-0.555
17	$CdAl_2Se_4$	-0.664	-0.665	-0.649	-0.441	-0.657	-0.457	**-0.6942**	-0.6929	-0.407	-0.624	-0.613	-0.393	-0.663	-0.542	-0.665	-0.547	-0.548	-0.550
18	$HgAl_2Se_4$	-0.507	-0.510	-0.506	0.127	-0.523	-0.353	**-0.5332**	-0.5302	-0.325	-0.499	-0.476	-0.324	-0.511	-0.400	-0.516	-0.428	-0.412	-0.426
19	$MgGa_2Se_4$	-0.702	-0.701	-0.610	-0.418	-0.602	-0.423	-0.7428	**-0.7445**	-0.358	-0.549	-0.550	-0.354	-0.670	-0.565	-0.668	-0.509	-0.563	-0.515
20	$CaGa_2Se_4$	-0.889	-0.887	-0.799	-0.444	-0.779	-0.583	-0.8943	**-0.8971**	-0.407	-0.756	-0.771	-0.507	-0.839	-0.734	-0.832	-0.687	-0.724	-0.690
21	$SrGa_2Se_4$	-0.857	-0.854	-0.767	-0.460	-0.747	-0.543	-0.8554	**-0.8584**	-0.462	-0.737	-0.464	-0.452	-0.803	-0.697	-0.797	-0.653	-0.687	-0.654
22	$ZnGa_2Se_4$	-0.452	-0.452	-0.373	-0.206	-0.380	-0.231	-0.5131	**-0.5138**	-0.167	-0.321	-0.302	-0.156	-0.440	-0.341	-0.445	-0.291	-0.349	-0.299
23	$CdGa_2Se_4$	-0.460	-0.459	-0.369	-0.075	-0.383	-0.222	-0.4882	**-0.4886**	-0.173	-0.348	-0.329	-0.160	-0.421	-0.322	-0.429	-0.288	-0.333	-0.294
24	$HgGa_2Se_4$	-0.307	-0.307	-0.237	0.011	-0.260	-0.118	-0.3330	**-0.3331**	-0.077	-0.233	-0.203	0.104	-0.275	-0.190	-0.290	-0.172	-0.207	-0.173
25	$MgAl_2Te_4$	-0.480	-0.480	-0.454	-0.246	-0.442	-0.254	-0.5252	**-0.5264**	-0.203	-0.394	-0.394	-0.202	-0.484	-0.365	-0.478	-0.341	-0.365	-0.350
26	$CaAl_2Te_4$	-0.695	-0.692	-0.667	-0.443	-0.640	-0.428	-0.7012	**-0.7047**	-0.239	-0.618	-0.638	-0.284	-0.675	-0.557	-0.664	-0.539	-0.547	-0.545
27	$SrAl_2Te_4$	-0.688	-0.685	-0.661	-0.156	-0.634	-0.415	-0.6866	**-0.6907**	-0.231	-0.623	-0.644	-0.255	-0.665	-0.546	-0.655	-0.531	-0.535	-0.536
28	$ZnAl_2Te_4$	-0.263	-0.264	-0.263	-0.097	-0.269	-0.113	**-0.3315**	-0.3307	-0.066	-0.214	-0.199	-0.071	-0.294	-0.187	-0.295	-0.175	-0.198	-0.180
29	$CdAl_2Te_4$	-0.300	-0.301	-0.279	-0.108	-0.289	-0.120	**-0.3366**	-0.3357	-0.088	-0.254	-0.235	-0.094	-0.300	-0.191	-0.303	-0.191	0.203	-0.194
30	$HgAl_2Te_4$	-0.185	-0.187	-0.202	0.269	-0.214	-0.069	**-0.2184**	-0.2162	-0.057	-0.189	-0.184	-0.065	-0.196	-0.115	-0.209	-0.134	-0.124	-0.130
31	$MgGa_2Te_4$	-0.372	-0.371	-0.267	-0.075	-0.267	-0.130	-0.4134	**-0.4148**	-0.114	-0.226	-0.219	-0.107	-0.336	-0.252	-0.336	-0.189	-0.254	-0.196
32	$CaGa_2Te_4$	-0.577	-0.575	-0.473	-0.299	-0.450	-0.281	-0.5801	**-0.5829**	-0.292	-0.276	-0.444	-0.265	-0.521	-0.433	-0.510	-0.376	-0.423	-0.378
33	$SrGa_2Te_4$	-0.567	-0.564	-0.471	-0.319	-0.444	-0.314	-0.5641	**-0.5674**	-0.296	-0.281	-0.453	-0.308	-0.512	-0.422	-0.499	-0.369	-0.410	-0.371
34	$ZnGa_2Te_4$	-0.161	-0.161	-0.099	0.116	-0.111	-0.001	-0.2267	**-0.2269**	0.016	-0.059	-0.056	0.031	-0.159	-0.093	-0.169	-0.041	-0.095	-0.041
35	$CdGa_2Te_4$	-0.193	-0.192	-0.110	0.058	-0.126	-0.003	-0.2279	**-0.2284**	0.022	-0.092	-0.081	0.026	-0.162	-0.096	-0.174	-0.051	-0.099	-0.051
36	$HgGa_2Te_4$	-0.083	-0.082	0.170	0.131	-0.056	0.056	**-0.1144**	-0.1143	0.080	-0.025	-0.024	0.073	-0.068	-0.019	-0.087	0.011	-0.020	0.017

Supplementary Table 7. Enthalpies of formation E_f (eV/atom) of γ_{1-3}- and δ_{1-9}-MA_2Z_4 monolayer materials, where M = Mg, Ca, Sr, Zn, Cd, Hg; A = Al and Ga; Z = S, Se and Te.

No.	Name	γ_1	γ_2	γ_3	δ_1	δ_2	δ_3	δ_4	δ_5	δ_6	δ_7	δ_8	δ_9
01	$MgAl_2S_4$	-0.984	-1.113	-0.977	-1.064	-0.932	-0.914	-1.102	-0.910	-1.067	-1.041	-0.784	-0.933
02	$CaAl_2S_4$	-1.122	-1.256	-1.123	-1.239	-0.705	-1.099	-1.277	-1.090	-1.243	-1.223	-0.509	-1.106
03	$SrAl_2S_4$	-1.055	-1.194	-1.052	-1.190	-0.692	-1.046	-1.228	-1.039	-1.192	-1.173	-0.467	-0.692
04	$ZnAl_2S_4$	-0.723	-0.850	-0.707	-0.794	-0.665	-0.637	-0.821	-0.645	-0.792	-0.771	-0.514	-0.662
05	$CdAl_2S_4$	-0.679	-0.810	-0.673	-0.785	-0.653	-0.638	-0.811	-0.650	-0.779	-0.772	-0.518	-0.648
06	$HgAl_2S_4$	-0.503	-0.629	-0.509	-0.613	-0.485	-0.479	-0.626	-0.505	-0.602	-0.611	-0.391	-0.481
07	$MgGa_2S_4$	-0.665	-0.774	-0.614	-0.732	-0.374	-0.548	-0.804	-0.548	-0.733	-0.661	-0.441	-0.374
08	$CaGa_2S_4$	-0.804	-0.918	-0.773	-0.904	-0.615	-0.488	-0.973	-0.735	-0.909	-0.850	-0.492	-0.619
09	$SrGa_2S_4$	-0.744	-0.862	-0.712	-0.859	-0.612	-0.469	-0.924	-0.692	-0.862	-0.809	-0.461	-0.613
10	$ZnGa_2S_4$	-0.414	-0.518	-0.349	-0.468	-0.364	-0.273	-0.527	-0.292	-0.462	-0.397	-0.189	-0.101
11	$CdGa_2S_4$	-0.371	-0.481	-0.320	-0.459	-0.148	-0.033	-0.514	-0.300	-0.450	-0.403	-0.025	-0.138
12	$HgGa_2S_4$	-0.204	-0.305	-0.160	-0.292	-0.188	0.090	-0.331	-0.157	-0.277	-0.248	0.092	0.010
13	$MgAl_2Se_4$	-0.794	-0.912	-0.785	-0.870	-0.752	-0.723	-0.907	-0.720	-0.874	-0.842	-0.603	-0.752
14	$CaAl_2Se_4$	-0.960	-1.080	-0.966	-1.065	-0.944	-0.939	-1.101	-0.926	-1.073	-1.050	-0.503	-0.948
15	$SrAl_2Se_4$	-0.919	-1.042	-0.924	-1.037	-0.913	-0.913	-1.071	-0.900	-1.044	-1.025	-0.464	-0.916
16	$ZnAl_2Se_4$	-0.567	-0.679	-0.546	-0.630	-0.517	-0.474	-0.654	-0.491	-0.628	-0.601	-0.380	-0.513
17	$CdAl_2Se_4$	-0.549	-0.665	-0.541	-0.642	-0.525	-0.500	-0.665	-0.516	-0.637	-0.626	-0.402	-0.521
18	$HgAl_2Se_4$	-0.410	-0.516	-0.421	-0.502	-0.393	-0.395	-0.510	-0.412	-0.492	-0.497	-0.325	-0.388
19	$MgGa_2Se_4$	-0.565	-0.666	-0.511	-0.632	-0.535	-0.450	-0.701	-0.453	-0.634	-0.558	-0.358	-0.532
20	$CaGa_2Se_4$	-0.726	-0.830	-0.699	-0.819	-0.598	-0.519	-0.888	-0.655	-0.828	-0.764	-0.415	-0.607
21	$SrGa_2Se_4$	-0.593	-0.796	-0.664	-0.792	-0.602	-0.497	-0.856	-0.634	-0.800	-0.745	-0.459	-0.606
22	$ZnGa_2Se_4$	-0.352	-0.443	-0.285	-0.401	-0.316	-0.225	-0.452	-0.241	-0.395	-0.327	-0.157	-0.302
23	$CdGa_2Se_4$	-0.334	-0.427	-0.278	-0.410	-0.318	-0.081	-0.460	-0.261	-0.402	-0.351	0.035	-0.154
24	$HgGa_2Se_4$	-0.208	-0.289	-0.164	-0.275	-0.192	-0.137	-0.308	-0.156	-0.260	-0.230	0.117	-0.172
25	$MgAl_2Te_4$	-0.367	-0.476	-0.344	-0.441	-0.337	-0.293	-0.480	-0.297	-0.443	-0.403	-0.199	-0.333
26	$CaAl_2Te_4$	-0.549	-0.663	-0.553	-0.650	-0.535	-0.334	-0.694	-0.507	-0.662	-0.629	-0.262	-0.543
27	$SrAl_2Te_4$	-0.537	-0.653	-0.545	-0.648	-0.531	-0.528	-0.686	-0.510	-0.660	-0.633	-0.244	-0.538
28	$ZnAl_2Te_4$	-0.199	-0.293	-0.176	-0.249	-0.161	-0.136	-0.264	-0.133	-0.240	-0.215	-0.065	-0.153
29	$CdAl_2Te_4$	-0.203	-0.302	-0.190	-0.279	-0.183	-0.164	-0.301	-0.168	-0.272	-0.254	-0.090	-0.173
30	$HgAl_2Te_4$	-0.122	-0.209	-0.132	-0.192	-0.106	-0.126	-0.187	-0.120	-0.172	-0.182	-0.061	-0.106
31	$MgGa_2Te_4$	-0.180	-0.333	-0.191	-0.310	-0.243	-0.116	-0.372	-0.159	-0.307	-0.230	-0.100	-0.240
32	$CaGa_2Te_4$	-0.378	-0.508	-0.385	-0.501	-0.379	-0.331	-0.576	-0.306	-0.512	-0.439	-0.297	-0.389
33	$SrGa_2Te_4$	-0.384	-0.498	-0.380	-0.495	-0.395	-0.326	-0.565	-0.310	-0.508	-0.444	-0.303	-0.402
34	$ZnGa_2Te_4$	-0.098	-0.166	-0.040	-0.133	-0.075	-0.010	-0.162	-0.013	-0.116	-0.061	0.033	-0.073
35	$CdGa_2Te_4$	-0.030	-0.171	-0.050	-0.156	-0.091	-0.030	-0.193	-0.028	-0.140	-0.090	0.032	-0.086
36	$HgGa_2Te_4$	-0.022	-0.085	0.013	-0.072	-0.008	0.022	-0.083	0.035	-0.047	-0.020	0.079	-0.007

Supplementary Table 8. Carrier mobility of α_2-WSi_2P_4, $2H$-MoS_2 and $2H$-WSe_2. Deformation potential E_1 (eV), in-plane stiffness C^{2D} (N/m), effective mass m^* (m_e), mobility μ (cm^2 V^{-1} s^{-1}) for electron (e) and hole (h) along a_{o1} (or zigzag) and a_{o2} (or armchair) directions (See **Supplementary Fig. 17**) at 300 K.

Compounds	carrier type	E_1	C^{2D}	m^*	μ
α_2-WSi_2P_4 (this work)	e(a_{o1})(K)	-10.90	227.49	0.43	147.22
	h(a_{o1})(K)	-6.75	227.49	0.35	466.70
	e(a_{o2})(K)	-10.71	227.74	0.43	152.66
	h(a_{o2})(K)	-6.82	227.74	0.35	457.67
MoS_2 (this work)	e(a_{o1})(K)	-11.24	132.04	0.46	70.22
	h(a_{o1})(K)	-5.68	132.04	0.59	169.45
	e(a_{o2})(K)	-11.25	132.74	0.46	70.37
	h(a_{o2})(K)	-5.70	132.74	0.59	168.91
WSe_2 (this work)	e(a_{o1})(K)	-10.95	119.52	0.34	119.76
	h(a_{o1})(K)	-4.92	119.52	0.47	321.87
	e(a_{o2})(K)	-10.29	119.77	0.35	130.53
	h(a_{o2})(K)	-5.03	119.77	0.47	309.92
MoS_2 (Ref 1)	e(a_{o1})(K)	-10.88	127.44	0.46	72.16
	h(a_{o1})(K)	-5.29	127.44	0.57	200.52
	e(a_{o2})(K)	-11.36	128.16	0.48	60.32
	h(a_{o2})(K)	-5.77	128.16	0.60	152.18
WSe_2 (Ref 2)	e(a_{o1})(K)	-10.23	121.10	0.35	135.08
	h(a_{o1})(K)	-4.65	121.10	0.46	373.65
	e(a_{o2})(K)	-10.71	120.80	0.33	—
	h(a_{o2})(K)	-4.52	120.80	0.44	—

Supplementary Table 9. Total energy (eV/unit) of the magnetic phases with respect to energy of the ferromagnetic phase. FM, AFM1, AFM2 and PM stand for the ferromagnetic, antiferromagnetic and paramagnetic phases, respectively. The configuration of AFM1 and AFM2 are shown in **Supplementary Fig. 18**.

Comp.	FM	AFM1	AFM2	PM	Comp.	FM	AFM1	AFM2	PM
α_1-VSi_2N_4	0.000	0.052	0.055	0.131	α_2-VSi_2As_4	0.000	0.059	0.058	0.082
α_1-$NbSi_2N_4$	0.000	0.003	0.003	0.003	α_2-VGe_2As_4	0.000	0.051	0.050	0.078
β_2-VGe_2N_4	0.000	0.074	0.075	0.103	β_2-$CrGe_2N_4$	0.000	0.565	0.658	3.580
α_1-$NbGe_2N_4$	0.000	0.017	0.018	0.020	β_1-$CrSi_2As_4$	0.000	0.017	0.017	0.576
α_1-$TaGe_2N_4$	0.000	0.001	0.001	0.001	β_1-$CrGe_2As_4$	0.000	0.047	0.047	0.719
δ_4- VSi_2P_4	0.000	0.039	0.039	0.048					
α_2-VGe_2P_4	0.000	0.032	0.032	0.040					

Supplementary Table 10. Space groups and band gaps of MZ$_2$ and A$_2$Z$_2$.

MZ_2	space group	band gap	ref.	MZ_2	space group	band gap	ref.	A_2Z_2	space group	band gap	ref.
TiN$_2$	P$\bar{3}$m1	—	4	MgS$_2$	N	—	—	Si$_2$N$_2$	P$\bar{6}$m2	1.74	4
ZrN$_2$	P$\bar{3}$m1	—	4	CaS$_2$	N	—	—	Ge$_2$N$_2$	P$\bar{6}$m2	1.17	4
HfN$_2$	P$\bar{3}$m1	—	4	SrS$_2$	N	—	—	Si$_2$P$_2$	P$\bar{6}$m2	1.52	4
TiP$_2$	P$\bar{3}$m1	—	4	ZnS$_2$	N	—	—	Ge$_2$P$_2$	P$\bar{6}$m2	1.35	4
ZrP$_2$	P$\bar{3}$m1	—	4	CdS$_2$	N	—	—	Si$_2$As$_2$	P$\bar{6}$m2	1.63	4
HfP$_2$	P$\bar{3}$m1	—	4	HgS$_2$	N	—	—	Ge$_2$As$_2$	P$\bar{6}$m2	1.20	3
TiAs$_2$	P$\bar{3}$m1	—	4	MgSe$_2$	N	—	—	Al$_2$S$_2$	P$\bar{6}$m2	2.033	3
ZrAs$_2$	P$\bar{3}$m1	—	4	CaSe$_2$	N	—	—	Ga$_2$S$_2$	P$\bar{6}$m2	2.477	3
HfAs$_2$	P$\bar{3}$m1	—	4	SrSe$_2$	N	—	—	Al$_2$Se$_2$	P$\bar{6}$m2	1.929	3
VN$_2$	N	—	—	ZnSe$_2$	N	—	—	Ga$_2$Se$_2$	P$\bar{6}$m2	1.893	3
NbN$_2$	P$\bar{6}$m2	—	5	CdSe$_2$	N	—	—	Al$_2$Te$_2$	P$\bar{6}$m2	1.784	3
TaN$_2$	N	—	—	HgSe$_2$	N	—	—	Ga$_2$Te$_2$	P$\bar{6}$m2	1.459	3
VP$_2$	N	—	—	MgTe$_2$	N	—	—	Al$_2$S$_2$	P$\bar{3}$m1	2.096	3
NbP$_2$	N	—	—	CaTe$_2$	N	—	—	Ga$_2$S$_2$	P$\bar{3}$m1	2.301	3
TaP$_2$	N	—	—	SrTe$_2$	N	—	—	Al$_2$Se$_2$	P$\bar{3}$m1	2.078	3
VAs$_2$	N	—	—	ZnTe$_2$	N	—	—	Ga$_2$Se$_2$	P$\bar{3}$m1	1.629	3
NbAs$_2$	N	—	—	CdTe$_2$	N	—	—	Al$_2$Te$_2$	P$\bar{3}$m1	2.039	3
TaAs$_2$	N	—	—	HgTe$_2$	N	—	—	Ga$_2$Te$_2$	P$\bar{3}$m1	1.375	3
CrN$_2$	C2/m	0.271	4	—	—	—	—	—	—	—	—
MoN$_2$	C2/m	1.257	4	—	—	—	—	—	—	—	—
WN$_2$	C2/m	0.526	4	—	—	—	—	—	—	—	—
CrP$_2$	P$\bar{1}$	—	4	—	—	—	—	—	—	—	—
MoP$_2$	P$\bar{1}$	0.027	4	—	—	—	—	—	—	—	—
WP$_2$	N	—	—	—	—	—	—	—	—	—	—
CrAs$_2$	P$\bar{1}$	—	4	—	—	—	—	—	—	—	—
MoAs$_2$	P$\bar{1}$	—	4	—	—	—	—	—	—	—	—
WAs$_2$	N	—	—	—	—	—	—	—	—	—	—
TiN$_2$	P$\bar{6}$m2	0.5	7	TiN$_2$	P$\bar{3}$m1	—	7	—	—	—	—
ZrN$_2$	P$\bar{6}$m2	0.8	7	ZrN$_2$	P$\bar{3}$m1	—	7	—	—	—	—
HfN$_2$	P$\bar{6}$m2	1.4	7	HfN$_2$	P$\bar{3}$m1	—	7	—	—	—	—
VN$_2$	P$\bar{6}$m2	—	7	VN$_2$	P$\bar{3}$m1	—	7	—	—	—	—
NbN$_2$	P$\bar{6}$m2	—	7	NbN$_2$	P$\bar{3}$m1	—	7	—	—	—	—
TaN$_2$	P$\bar{6}$m2	0.5	7	TaN$_2$	P$\bar{3}$m1	—	7	—	—	—	—
CrN$_2$	P$\bar{6}$m2	—	7	CrN$_2$	P$\bar{3}$m1	—	7	—	—	—	—
MoN$_2$	P$\bar{6}$m2	–	6,7	MoN$_2$	P$\bar{3}$m1	—	7	—	—	—	—
WN$_2$	P$\bar{6}$m2	—	7	WN$_2$	P$\bar{3}$m1	0.3	7	—	—	—	—

[1] Y. Cai, G. Zhang, and Y.-W. Zhang, J. Am. Chem. Soc. **136**, 6269 (2014).

[2] H. L. Zhuang and R. G. Hennig, Chem. Mater. **25**, 3232 (2013).

[3] J. Zhou, L. Shen, M. D. Costa, K. A. Persson, S. P. Ong, P. Huck, Y. Lu, X. Ma, Y. Chen, H. Tang, Y. P. Feng, Scientific Data. **6**, 1C10 (2019).

[4] B. Özdamar, G. Özbal, M. N. Çınar, K. Sevim, G. Kurt, B. Kaya, and H. Sevinçli, Phys. Rev. B **98**, 045431 (2018).

[5] X. Guo, F. Li, Y. Zhu, X. Han, Y. Yan, Appl. Phys. Lett. **116**, 082403 (2020).

[6] Wang, Y., Wang, S.-S., Lu, Y., Jiang, J. and Yang, S. A., Nano Lett. **16**, 4576C4582 (2016).

[7] Liu, J., Liu, Z., Song, T. and Cui, X., J. Mater. Chem. C 5, 727C732 (2017).
